# Muscle toxicity reports in FAERS: a disproportionality analysis with focus on rhabdomyolysis and cross-database assessment

**DOI:** 10.3389/fphar.2026.1852621

**Published:** 2026-06-22

**Authors:** Lei Zhang, Yuqi Wang, Fang Wang, Kaiyun Ji, Lili Yang, Jia Li

**Affiliations:** 1 Department of Pharmacy, The First Affiliated Hospital of Sun Yat-sen University, Guangzhou, China; 2 Department of Pharmacy, Shanxi Provincial Integrated TCM and WM Hospital, Taiyuan, China; 3 Department of Pharmacy, Guizhou Hospital of The First Affiliated Hospital of Sun Yat-sen University (The Affiliated Hospital of Guizhou Medical University), Guiyang, China; 4 Department of Pharmacy, Jincheng General Hospital, Jincheng, China; 5 Department of Pharmacy, Guangxi Hospital Division of the First Affiliated Hospital, Sun Yat-sen University, Nanning, China

**Keywords:** adverse events, disproportionality analysis, FAERS, muscle toxicity, rhabdomyolysis

## Abstract

**Objective:**

Muscle toxicity can significantly impair quality of life and may be life-threatening in severe cases. Although statins are well known for their risk of muscle toxicity, a comprehensive evaluation of other implicated drugs remains limited. This study aimed to systematically characterize drug-related muscle toxicity using adverse event (AE) reports from the U.S. Food and Drug Adverse Event Reporting System (FAERS).

**Methods:**

FAERS data from the first quarter of 2004 to the fourth quarter of 2024 were extracted and processed. Signal detection was conducted using three disproportionality analysis methods: Reporting Odds Ratio (ROR), Proportional Reporting Ratio (PRR), and Bayesian Confidence Propagation Neural Network (BCPNN). Sensitivity analyses were conducted through stratification by sex, age, and reporter type. External validation was performed using the WHO VigiAccess database.

**Results:**

A total of 49,289 reports related to muscle toxicity were identified, involving 47,241 cases and 220 drugs. The mean time to onset was 324.75 days, with a median of 30.00 days. Nervous system drugs accounted for the largest proportion (27.3%), followed by anti-infective agents (22.3%), and cardiovascular drugs (19.5%). The most frequently reported drugs included atorvastatin (n = 5,275), simvastatin (n = 4,831), rosuvastatin (n = 3,209), levetiracetam (n = 1,021), and quetiapine (n = 725). Several drugs not prominently described for muscle toxicity in product labeling showed disproportionality signals, including furosemide [n = 239; ROR (95%CI):3.35 (2.95–3.81)], and diazepam [n = 141; ROR (95%CI): 2.78 (2.36–3.28)]. Rhabdomyolysis was the most frequently reported and clinically significant AE. Additional signals were observed for drugs with limited or unclear labeling regarding rhabdomyolysis, including oseltamivir [n = 63; ROR (95%CI): 2.51 (1.96–3.22)], metformin [n = 397; ROR (95%CI): 2.52 (2.28–2.78)], and alprazolam [n = 178; ROR (95%CI): 2.60 (2.24–3.01)]. Sensitivity analyses and external validation showed generally consistent patterns across subgroups and databases.

**Conclusion:**

This study provides a comprehensive pharmacovigilance evaluation of muscle toxicity–related reports and corresponding drugs using FAERS data. Several drugs with signals not prominently described in current product labeling were identified. These findings highlight the importance of continued pharmacovigilance and may support signal detection and hypothesis generation for future research.

## 1 Introduction

Drug-induced muscle toxicity is an important adverse drug reaction that encompasses a broad clinical spectrum, ranging from asymptomatic creatine kinase elevation and myalgia to myopathy and severe rhabdomyolysis (Steinmeyer and Flechtenmacher, 2023). Among these manifestations, rhabdomyolysis is considered one of the most serious forms because it may result in acute kidney injury, electrolyte disturbances, prolonged hospitalization, and death (Baeza-Trinidad, 2022). Early identification of drugs potentially associated with muscle toxicity is therefore of considerable importance in both clinical practice and post-marketing drug safety surveillance.

Although statins are widely recognized as major contributors to drug-associated muscle toxicity (Stroes et al., 2015), increasing evidence suggests that a broad range of other medications, including anti-infective agents, psychotropic drugs, corticosteroids, and immunomodulatory therapies, may also be associated with muscle-related adverse events (AEs) (Touat et al., 2018; Coutinho et al., 2023; AlKhateeb et al., 2025). However, these AEs are often difficult to recognize in routine clinical settings because their manifestations may be nonspecific and influenced by multiple confounding factors, including underlying diseases, renal dysfunction, infections, overdose, immobilization, and polypharmacy. In particular, drug-drug interactions may further increase the risk of severe muscle toxicity and rhabdomyolysis (Zhang et al., 2024).

Pharmacovigilance databases provide valuable real-world resources for the early detection of potential drug safety signals. The U.S. Food and Drug Administration Adverse Event Reporting System (FAERS), one of the largest spontaneous reporting systems worldwide, has been widely used for signal detection and post-marketing safety assessment (Wu et al., 2025). Nevertheless, most previous pharmacovigilance studies on muscle toxicity have focused on individual drugs or limited drug-event pairs, while comprehensive evaluations of broader drug classes, severe rhabdomyolysis-related events, and potential drug-drug interaction signals remain limited.

Therefore, the present study aimed to systematically characterize reporting signals associated with drug-induced muscle toxicity using FAERS data, with particular emphasis on rhabdomyolysis-related events. Multiple disproportionality methods, stratified analyses, drug-drug interaction signal exploration, supplementary cross-database consistency assessments, and product labeling reviews were conducted to provide a more comprehensive evaluation of potential reporting associations related to muscle toxicity in real-world pharmacovigilance data.

## 2 Methods

### 2.1 Data source and data processing

**FIGURE 1:**
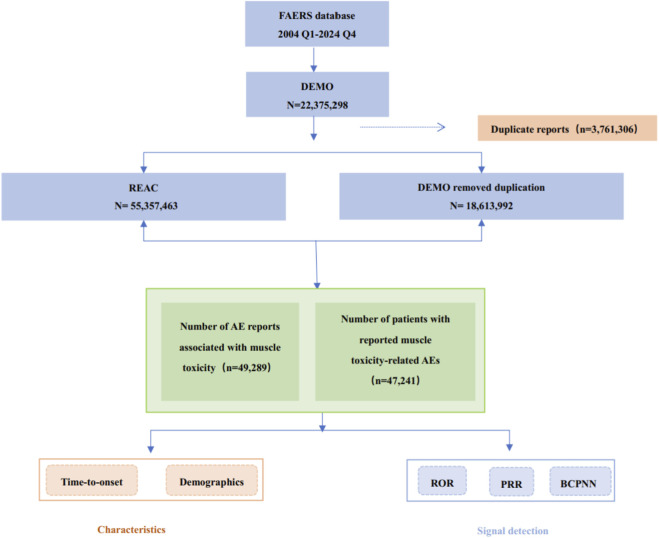
Flowchart of FAERS data extraction, cleaning, and analysis for muscle toxicity. Notes: “DEMO” refers to the FAERS demographic and administrative information table, in which N represents the number of reports. “REAC” refers to the adverse reaction table containing PTs, in which N represents the number of recorded adverse reaction entries. “DEMO after deduplication” represents deduplicated patient-level data. “Number of AE reports” refers to report-level data, whereas “Number of patients with reported muscle toxicity–related AEs” refers to deduplicated patient-level data.

Data for this study were obtained from the publicly available FAERS database (https://fis.fda.gov/extensions/FPD-QDE-FAERS/FPD-QDE-FAERS.html), which has been accessible since the first quarter of 2004 (Q1 2004) and is updated quarterly. FAERS contains spontaneous reports of adverse events (AEs), medication errors, and product quality complaints submitted by healthcare professionals, consumers, and manufacturers (Duggirala et al., 2016), comprising over 21 million reports to date. Data from Q1 2004 to the fourth quarter of 2024 (Q4 2024) were extracted from the FAERS database. Data cleaning and statistical analyses were performed using SAS version 9.4. The data preprocessing workflow is shown in Figure 1.

AEs in FAERS were coded using the Medical Dictionary for Regulatory Activities (MedDRA, version27.1). Preferred Terms (PTs) were used to identify specific AEs. Standardized MedDRA Queries (SMQs) were applied to group related PTs into clinically relevant categories (Xing et al., 2025). Drug names were standardized according to the World Health Organization (WHO) Drug Dictionary (September 2024 version). Drugs were classified according to the Anatomical Therapeutic Chemical (ATC) classification system developed by WHO, and grouped at the first-level ATC category. For drugs associated with multiple ATC codes, each drug was assigned to a single first-level ATC category according to its predominant approved therapeutic indication to avoid duplicate counting across categories. Combination products were analyzed as independent drug entities and were not decomposed into individual active ingredients. Each combination product was assigned to one first-level ATC category based on its predominant therapeutic use. Only drugs coded as primary suspect (PS) in the FAERS database were included, to reduce confounding from concomitant medications and allow more specific signal detection. To explore associations between drug exposure and muscle toxicity, this study utilized the narrow scope of the SMQ “rhabdomyolysis/myopathy”, which initially included 15 PTs related to muscle toxicity (see Supplementary Table S1). Four PTs (muscle infarction, thyrotoxic myopathy, hypothyroid myopathy, and diabetic myonecrosis) were excluded from downstream analyses, because their underlying etiologies are more commonly associated with ischemic, endocrine, or metabolic disorders rather than drug-induced muscle toxicity. Consequently, all subsequent analyses were conducted using the final set of 11 PTs.

### 2.2 Statistical analysis

This study was performed according to the reporting of a disproportionality analysis for drug safety signal detection using individual case safety reports in pharmacovigilance (READUS-PV) (Fusaroli et al., 2024). Three disproportionality methods were applied to detect potential AE signals in the FAERS database: reporting odds ratio (ROR), proportional reporting ratio (PRR), Bayesian confidence propagation neural network (BCPNN). Detailed formulas and signal detection criteria are provided in Supplementary Tables S2, S3.

The ROR method assesses the likelihood of an AE reported for a specific drug compared with all other drugs. A signal is considered positive if the number of reports (*a* value)≥3, and the lower limit of 95% Confidence Interval (CI) for the ROR>1 (Ooba and Kubota, 2010). The PRR method compares the proportion of a specific AE for a drug with the proportion of the same AE for all other drugs. A signal is detected when the number of reports (*a* value) is ≥ 3, the chi-square statistic (*χ* ^*2*^) > 4 and the PRR value > 2 (Evans et al., 2001); The BCPNN method uses Bayesian inference to calculate the information component (IC), and a signal is considered significant if the lower limit of the 95% CI of the IC (IC025) is greater than 0 and the number of reports (*a* value)≥3 (Bate et al., 1998).

In pharmacovigilance, ROR and PRR are widely used for disproportionality analysis due to their simplicity and ease of interpretation. However, both methods may be limited by variability in spontaneous reporting rates, which may affect the accuracy of signal detection. In contrast, the BCPNN adjusts for such variability and produces more stable estimates, especially when the data are sparse.

To improve the robustness and specificity of signal detection, a positive signal was defined only when all three algorithms (ROR, PRR, and BCPNN) simultaneously met their respective signal detection thresholds. This combined approach may reduce false-positive findings; however, it may also reduce sensitivity for rare drug–event pairs with small numbers of reports.

For descriptive analyses of demographic characteristics, duplicate reports were removed and each unique CASEID was considered as a single case. Disproportionality analyses were conducted using the deduplicated dataset, with each case counted only once to avoid artificial signal amplification caused by repeated submissions of the same event.

To further evaluate potential drug–drug interaction (DDI) effects associated with muscle toxicity, DDI signal detection was performed using the Ω shrinkage measure. The Ω statistic quantifies the deviation between the observed and expected reporting frequency of a target AE following combined exposure to two drugs. A positive DDI signal was defined when the lower limit of the 95% CI of Ω (Ω025) exceeded 0, indicating a potential synergistic interaction associated with the target AE (Norén et al., 2008). Detailed formulas and calculation principles for the Ω shrinkage measure are provided in Supplementary Table S4. All statistical analyses were performed using SAS 9.4 software and Microsoft EXCEL 365.

### 2.3 Time to onset analysis

Time to onset (TTO) was defined as the interval (in days) between the drug start date and the AE onset date. Reports with missing or logically inconsistent dates (e.g., event onset preceding drug initiation) were excluded from the TTO analysis. Zero-day TTO values were retained because they may represent AEs occurring on the same day as drug initiation. To evaluate the influence of extreme values on the TTO distribution, sensitivity analyses were performed by excluding observations above the 99th percentile (P99) of the TTO distribution. Descriptive statistics were summarized using medians and interquartile ranges (IQRs) because of the skewed distribution of TTO data.

### 2.4 Sensitivity analysis

To assess the robustness of the findings, subgroup analyses were conducted according to sex, age, and reporter type. Sex was categorized as male, female and not specified. Age was grouped into four categories: <18 years, 18–44 years, 45–64 years, and ≥65 years, based on the available information in the FAERS database. In addition, analyses restricted to reports submitted by healthcare professionals were performed to examine the consistency of the results across different reporting sources. Furthermore, to assess the stability of low-count signals, sensitivity analyses were performed using minimum report thresholds of ≥10, ≥20, and ≥50 reports. Signals based on fewer than 10 reports were considered exploratory and interpreted cautiously because of potential statistical instability and wide confidence intervals.

Disproportionality analyses were repeated within each subgroup using the same methods and criteria as in the primary analysis to evaluate the consistency of the detected signals. In addition, the Multi-item Gamma Poisson Shrinker (MGPS) method was used as a supplementary analysis to examine whether the major positive signals identified in the primary analyses showed similar patterns under an alternative disproportionality approach. MGPS signal detection was based on the empirical Bayes geometric mean (EBGM), and signals were considered present when the lower 5% confidence limit (EBGM05) exceeded 2. MGPS was used solely as a supplementary robustness assessment and was not included in the primary signal definition criteria. Primary positive signals were defined based on concordant positive results from the ROR, PRR, and BCPNN analyses, whereas EBGM and EBGM05 values were additionally calculated to provide supportive evidence regarding the consistency of the detected signals. The detailed formulas and signal detection criteria for MGPS are provided in Supplementary Table S3.

### 2.5 Cross-database assessment

To assess the consistency of reporting patterns across pharmacovigilance databases, a supplementary cross-database disproportionality assessment was conducted using aggregated reporting data from the WHO VigiAccess database. Positive signals identified in FAERS were compared with corresponding reporting patterns observed in VigiAccess to evaluate cross-database consistency. WHO VigiAccess is a publicly accessible portal of the WHO Programme for International Drug Monitoring maintained by the Uppsala Monitoring Centre and provides aggregated spontaneous reporting data coded using the MedDRA terminology. Data were retrieved from the VigiAccess database (https://www.vigiaccess.org) on 29 December 2024. To facilitate large-scale aggregated data retrieval, Python 3.10 was used to access front-end JSON data through the requests package. BASENAME terms from the WHO Drug Dictionary were used for drug identification and mapping. Retrieved JSON data were processed and structured using the pandas package and subsequently exported to Excel for data management and statistical analysis. The same PT definitions, SMQ framework, disproportionality algorithms, signal thresholds, and 2 × 2 contingency table construction methods used in the FAERS analysis were directly applied to the VigiAccess data to ensure methodological consistency between the two databases. Statistical analyses were performed using SAS version 9.4.

### 2.6 Literature search

A structured literature search was performed to identify published evidence related to drug-associated muscle toxicity and to provide contextual support for the findings of this study. The relevant articles were retrieved from PubMed, Embase, and Web of Science from database inception to March 2026. The search strategy combined terms related to muscle toxicity, including “muscle toxicity”, “myopathy”, and “rhabdomyolysis”, using appropriate Boolean operators. Studies were eligible for inclusion if they reported muscle toxicity-related AEs associated with drug exposure. Randomized controlled trials (RCTs), observational studies, and case reports were considered. Titles and abstracts were screened, and relevant full-text articles were reviewed. Data on reported drugs, associated AEs, and key findings were extracted and summarized. The extracted information is presented in Table 1.

**TABLE 1:** Summary of published studies on drug-associated muscle toxicity.

Study type	Author.year	Intervention	Related AEs	Outcome
Retrospective observational study	Chen et al. (2025)	Colesevelam	Musculoskeletal toxicity	Myalgia: ROR4.74, 95%CI 3.89–5.78 muscle spasms: ROR 3.43, 95%CI 2.74–4.29
Retrospective observational study	Sun et al. (2024)	PPI	Rhabdomyolysis	Lansoprazole: ROR 12.67, 95%CI 11.66–13.77 Esomeprazole: ROR11.18; 95%CI 10.38–12.05 Rabeprazole: ROR = 10.06; 95%CI 8.43–12.02
Retrospective observational study	Capogrosso Sansone et al. (2017)	PPI	Muscle toxicity–related ADRs	Muscle toxicity–related ADRs Esomeprazole: ROR 2.062; 95%CI 1.593–2.669 Lansoprazole: ROR 1.186, 95%CI 0.918–1.531 Omeprazole: ROR = 1.302; 95%CI 1.031–1.643 Pantoprazole: ROR = 1.154; 95%CI 0.896–1.487 Rabeprazole: ROR = 1.668; 95%CI 1.044–2.663 Rhabdomyolysis Lansoprazole: ROR 2.050, 95%CI 1.140–3.688 Omeprazole: ROR = 2.142; 95%CI 1.244–3.690
Retrospective observational study	Guo et al. (2025)	Levetiracetam	Rhabdomyolysis	ROR 13.52, 95%CI 12.89–14.14
Retrospective observational study	Yin et al. (2025)	Quetiapine	Rhabdomyolysis	ROR 3.81, 95%CI 0.53–27.6
Case report	Sharma et al. (2023)	Valproate	Rhabdomyolysis	Rhabdomyolysis following Valproate use, resolved after discontinuation
Retrospective observational study	Ohyama et al. (2025)	SSRIs	Rhabdomyolysis	Sertraline: aROR1.76, 95%CI 1.14–2.72 Escitalopram: aROR1.86, 95%CI 1.07–3.23
Case report	Sathiavageesan and Murugan (2023)	Tramadol, ondansetron	Rhabdomyolysis	Suspected rhabdomyolysis possibly caused by valproic acid or when it is combined with ondansetron
Retrospective observational study	Teng et al. (2019)	Azithromycin, linezolid	Rhabdomyolysis	Azithromycin: ROR 2.94, 95%CI 1.96–4.39 Linezolid: ROR 2.49, 95%CI 1.47–4.21
Retrospective observational study	Tian et al. (2025)	Voriconazole	Rhabdomyolysis	Rhabdomyolysis is a new ADE signal of voriconazole
Case report	Puttagunta et al. (2018)	Oseltamivir	Rhabdomyolysis	Suspected oseltamivir-induced rhabdomyolysis, the Naranjo algorithm applied gave a score of 3
Case report	Wrafter et al. (2020)	losartan	Rhabdomyolysis	Suspected losartan-induced rhabdomyolysis
Retrospective observational study	Liu et al. (2024)	Irbesartan	Rhabdomyolysis	ROR 7.76, 95%CI 5.88–10.25
Case report	El Bahri et al. (2025)	Amiodarone	Uncommon neuromyopathy toxicity	Uncommon neuromyopathy toxicity following amiodarone use, resolved after discontinuation
Retrospective observational study	He et al. (2024)	Diazepam	Rhabdomyolysis, Blood creatine phosphokinase increased, Hypotonia,Myoclonus, Muscle rigidity	Rhabdomyolysis: ROR3.18, 95%CI 2.64–3.84 Blood creatine phosphokinase increased: ROR 2.58, 95%CI 2.04–3.28 Hypotonia: ROR7.88, 95%CI 6.21–9.98 Myoclonus: ROR 3.88, 95%CI 2.83–5.32 Muscle rigidity: ROR 3.31, 95%CI 2.35–4.67

aROR: adjusted reporting odds ratio; PPI: proton pump inhibitor; SSRIs:selective serotonin reuptake inhibitors.

### 2.7 Definition of analytical units

In this study, two types of analytical units were defined and applied according to the purpose of each analysis, as specified below.

#### 2.7.1 Case-level unit

Case-level analyses were performed using deduplicated patients according to the FDA-recommended FAERS duplicate-report handling procedures. Each patient was counted only once regardless of the number of reports or AEs recorded. This unit was used for descriptive analyses of demographic characteristics (e.g., age, sex, and reporting country) and clinical outcomes (e.g., death, hospitalization, and disability).

#### 2.7.2 Report-level unit

Report-level analyses were performed using individual FAERS reports. Each report was counted separately, even if multiple reports were linked to the same patient. This unit was used for disproportionality analyses, including ROR, PRR, BCPNN, and MGPS analyses. Signal detection analyses were conducted based on drug–event pairs identified within reports.

### 2.8 Drug labeling review

A systematic review of publicly available official drug labeling documents was conducted to identify information related to rhabdomyolysis, myopathy, and severe muscle toxicity. Labeling documents were retrieved from official regulatory databases using drug names standardized according to the WHO Drug Dictionary (September 2024 version). The primary data sources included the U.S. FDA DailyMed database and the European Medicines Agency (EMA) Summary of Product Characteristics (SmPCs). For drugs approved in multiple regions, the most recent official labeling document from FDA DailyMed or EMA SmPCs was used for evaluation.

All labeling assessments were independently performed by two authors (WF and JKY) using a standardized data extraction framework. Labeling information was categorized into four predefined groups: D, direct warnings or precautions related to rhabdomyolysis or muscle toxicity; PM, postmarketing reports describing rhabdomyolysis or muscle toxicity; DDI, drug-drug interaction warnings associated with increased risk of rhabdomyolysis or muscle toxicity; and N, no explicit mention of rhabdomyolysis or muscle toxicity in the official labeling documents. Discrepancies between the two reviewers were resolved through consensus discussion, and a third senior author (LJ) was consulted when necessary. In the present study, a labeling gap was defined as the absence of direct warnings or precautions regarding rhabdomyolysis or muscle toxicity in the official labeling documents. This standardized review procedure was implemented to improve the transparency, consistency, and reproducibility of the labeling assessment.

## 3 Results

### 3.1 Descriptive analysis

**FIGURE 2:**
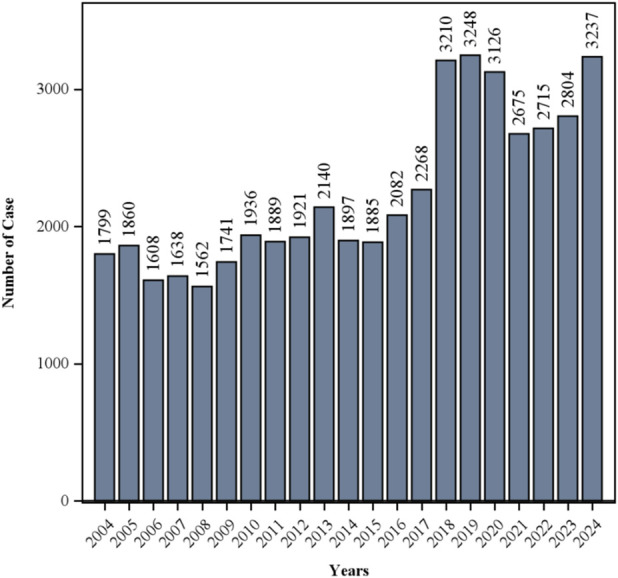
Annual number of reported muscle toxicity cases in the FAERS from 2004 to 2024. Notes: Case counts were based on deduplicated patient-level data.

In this study, a total of 55,357,463 AE reports involving 18,613,992 patients were obtained from the FAERS database after data cleaning and deduplication, covering the period from Q1 2004 to Q4 2024. Among these, 49,289 muscle toxicity–related reports involving 47,241 deduplicated patients were identified (Figure 1). The demographic characteristics of these cases were summarized in Table 2, Figure 2. A greater proportion of cases involved males (n = 25,518; 54.02%) compared with females (n = 16,978; 35.94%). The largest proportion of cases was observed among individuals aged ≥65 years (n = 15,314; 32.42%). The majority of reports were submitted by physicians (n = 18,412; 38.97%) and pharmacists (n = 10,756; 22.77%). The largest proportion of reports originated from the United States (n = 14,179; 30.01%), followed by France (n = 4,665; 9.87%) and Japan (n = 4,426; 9.37%). These data are detailed in Supplementary Table S5. The annual number of reports related to muscle toxicity showed a gradual increase from 2004 to 2024, reaching a peak of 3,248 cases in 2019. The yearly trend is illustrated in Figure 2, showing an overall upward pattern over the study period.

**TABLE 2:** Demographic and clinical characteristics of muscle toxicity reports from the FAERS database.

Characteristics	Cases, n (%) (total cases:47241)
Sex	
Female	16978 (35.94)
Male	25518 (54.02)
Not specified	4745 (10.04)
Age	
<18y	1710 (3.62)
18–44y	8371 (17.72)
45–64y	12490 (26.44)
≥65y	15314 (32.42)
Not specified	9356 (19.80)
Continent	
Europe	18910 (40.03)
North America	16003 (33.88)
Asia	7169 (15.18)
Not specified	3620 (7.66)
Oceania	1069 (2.26)
South America	286 (0.61)
Africa	184 (0.39)
Reporter	
Consumer	4558 (9.65)
Lawyer	230 (0.49)
Not specified	3003 (6.36)
Other health-professional	10282 (21.76)
Pharmacist	10756 (22.77)
Physician	18412 (38.97)
Outcomes	
Life-threatening	7132 (15.10)
Hospitalization - initial or prolonged	29966 (63.43)
Disability	2898 (6.13)
Death	4668 (9.88)
Congenital Anomaly	38 (0.08)
Required intervention to prevent permanent impairment/Damage	868 (1.84)
Other	27133 (57.44)

n represents the number of deduplicated patients with muscle toxicity–related AEs (case-level unit). Percentages were calculated using 47,241 patients as the denominator.

The distribution of TTO showed that the largest number of cases occurred within 30 days after drug initiation (n = 7,084; 15.00%). The median TTO was 30.00 days (IQR: 4.00–220.00 days), and the mean TTO was 324.75 days. The observed TTO ranged from 0 to 18,113 days. The detailed TTO distribution is presented in Supplementary Figure S1.

Among the 47,241 cases analyzed, 46,378 (98.17%) were reported as severe outcomes. The most commonly reported outcomes included hospitalization (63.43%), life-threatening events (15.10%), death (9.88%), disability (6.13%), congenital anomalies (0.08%), and intervention required to prevent permanent damage (1.84%). Additionally, 27,133 cases (57.44%) were classified as other medically important events (Table 2). Because outcome variables in FAERS are recorded in a multiple-response format, the total number of reported outcomes exceeds the number of individual cases.

### 3.2 Disproportionality analysis

#### 3.2.1 Distribution of drugs with positive signals across therapeutic categories

**FIGURE 3:**
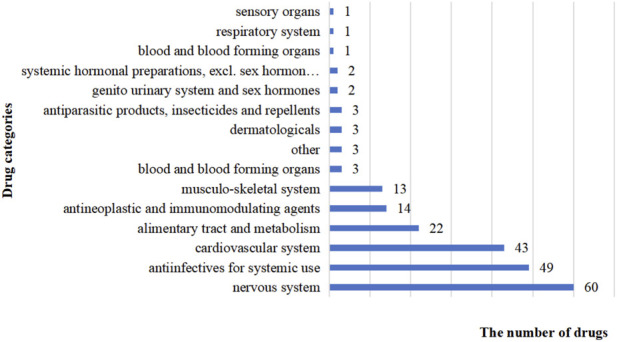
Number of drugs with positive signals across ATC therapeutic categories in FAERS. Notes: Drug counts were based on unique drugs with at least one positive signal within each ATC therapeutic category.

A total of 220 drugs showed positive signals for muscle toxicity in the FAERS database, the list of 220 drugs and the corresponding signals is presented in Supplementary Table S6. Figure 3 presents the distribution of these drugs across therapeutic categories. Neurological agents accounted for the largest proportion (n = 60; 27.3%), followed by anti-infective agents (n = 49; 22.3%) and cardiovascular drugs (n = 43; 19.5%).

#### 3.2.2 Distribution of drugs with positive signals at PT level

**FIGURE 4:**
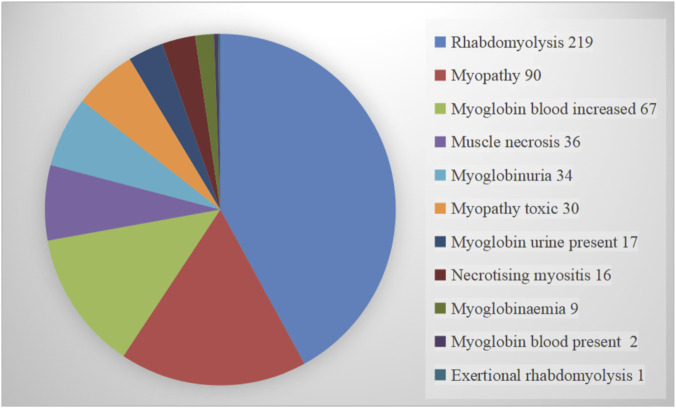
Distribution of drugs with positive signals across PTs within the SMQ “rhabdomyolysis/myopathy”. Notes: Drug counts were summarized according to PT classification. Because a single drug could be associated with more than one PT, the total number of drug–PT pairs exceeded the number of unique drugs included in the analysis.

Drugs with positive signals identified within the narrow scope of the SMQ “rhabdomyolysis/myopathy” were categorized according to their corresponding PTs, and the number of drugs associated with each PT was summarized. As shown in Figure 4, rhabdomyolysis accounted for the largest number of drugs with positive signals (n = 219), representing 42.03% of all identified drug-PT pairs. Because a single drug may be associated with more than one PT, the total number of drug-PT pairs exceeds the number of unique drugs included in the analysis (n = 220).

#### 3.2.3 Top 50 drugs by reporting frequency of muscle toxicity

**FIGURE 5:**
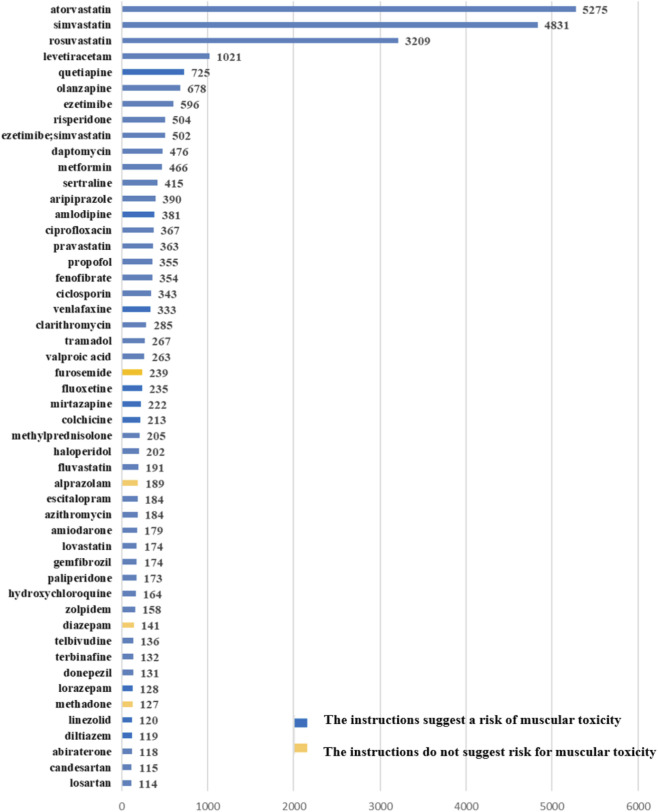
Top 50 most frequently reported drugs associated with muscle toxicity. Notes: The horizontal axis represents the number of muscle toxicity–related reports associated with each drug.

The top 50 most frequently reported drugs associated with muscle toxicity are shown in Figure 5. Atorvastatin accounted for the highest number of reports (n = 5,275), followed by simvastatin (n = 4,831), rosuvastatin (n = 3,209), levetiracetam (n = 1,021), quetiapine (n = 725), olanzapine (n = 678), ezetimibe (n = 596), risperidone (n = 504), the ezetimibe/simvastatin combination (n = 502), and daptomycin (n = 476). Among these agents, those acting on cardiovascular system (21 drugs; 42%), nervous system (17 drugs; 34%), and anti-infective agents (6 drugs; 12%) were most frequently represented. Notably, for several drugs in this list, muscle toxicity is not prominently described in current product labeling, including furosemide, amlodipine, diazepam, methadone.

#### 3.2.4 Top 50 drugs ranked by ROR signal intensity for muscle toxicity

Table 3 lists the top 50 drugs ranked by ROR signal intensity for muscle toxicity. The top five drugs were sodium nitrite, bezafibrate, simvastatin, lovastatin and gemfibrozil. Among these drugs, cardiovascular agents were the most represented (n = 16; 32%), followed by neurological drugs (n = 9; 18%), anti-infective agents (n = 7; 14%), digestive and metabolic drugs (n = 6; 12%), and musculo-skeletal system drugs (n = 5; 10%). For several drugs in this list, muscle toxicity is not prominently described in current product labeling, including delorazepam, peramivir, and the combination of alogliptin and metformin.

**TABLE 3:** The top 50 drugs associated with muscle toxicity ranked according to ROR signal intensity.

Drug name	Case reports	ROR (95% CI)	PRR (χ^2^)	IC(IC025)	Package insert suggests risk for muscle toxicity	Time to event (median (Q1-Q3)) (days)
Sodium nitrite	3	153.03 (45.80,511.29)	134.78 (398.70)	7.07 (0.42)	N	0.00 (0.00,0.00)
Bezafibrate	3	105.21 (32.21,343.58)	96.27 (283.09)	6.59 (0.43)	Y	1.50 (0.00,3.00)
Simvastatin	4831	61.68 (59.84,63.58)	58.81 (247,850)	5.73 (5.67)	D	245.00 (35.00,1035.00)
Lovastatin	174	54.45 (46.76,63.42)	51.99 (8678.41)	5.70 (5.10)	D	199.00 (32.00,601.00)
Gemfibrozil	174	53.50 (45.94,62.31)	51.12 (8527.93)	5.67 (5.08)	DDI	109.00 (30.00,517.00)
colchicine; probenecid	29	51.93 (35.78,75.36)	49.68 (1383.69)	5.63 (3.70)	D	​
ezetimibe; simvastatin	502	51.85 (47.39,56.73)	49.62 (23,695.1)	5.62 (5.35)	D	85.00 (26.00,219.00)
Triheptanoin	52	51.15 (38.73,67.54)	48.96 (2442.77)	5.61 (4.28)	N	247.00 (138.00,1053.00)
Telbivudine	136	41.82 (35.23,49.64)	40.36 (5210.22)	5.33 (4.72)	Y	302.50 (257.00,389.00)
Cerivastatin	8	32.77 (16.22,66.19)	31.87 (239.36)	4.99 (1.87)	Y	16.50 (0.00,33.00)
Fluvastatin	191	28.71 (24.87,33.16)	28.03 (4962.83)	4.80 (4.40)	D	107.00 (21.00,582.00)
Trabectedin	101	28.10 (23.06,34.24)	27.44 (2570.21)	4.78 (4.15)	D	28.00 (9.50,48.00)
Rosuvastatin	3209	27.84 (26.85,28.87)	27.23 (75,870.0)	4.67 (4.61)	D	94.00 (23.00,344.00)
Atorvastatin	5275	27.47 (26.68,28.27)	26.90 (117,556)	4.59 (4.54)	D	94.00 (22.00,681.00)
Daptomycin	476	26.06 (23.79,28.55)	25.50 (11,105.9)	4.66 (4.45)	D	9.00 (4.00,13.00)
atorvastatin; ezetimibe	36	25.95 (18.64,36.11)	25.38 (843.35)	4.66 (3.45)	D	52.00 (27.50,146.50)
amoxicillin; clarithromycin; esomeprazole	3	25.90 (8.24,81.35)	25.34 (70.18)	4.66 (0.37)	Y	​
Delorazepam	3	24.22 (7.72,76.02)	23.73 (65.37)	4.57 (0.36)	N	0.00 (0.00,0.00)
Fenofibrate	354	23.37 (21.03,25.97)	22.92 (7373.28)	4.51 (4.27)	D	56.50 (11.00,333.00)
Peramivir	10	22.27 (11.91,41.64)	21.85 (199.14)	4.45 (2.04)	N	1.00 (1.00,2.50)
Pitavastatin	99	21.67 (17.76,26.44)	21.28 (1911.26)	4.41 (3.85)	D	118.00 (19.00,274.00)
Pravastatin	363	21.54 (19.41,23.91)	21.16 (6927.16)	4.39 (4.16)	D	138.00 (52.00,678.00)
Maprotiline	19	20.95 (13.31,32.99)	20.59 (354.23)	4.36 (2.73)	N	104.50 (5.00,2443.00)
Colchicine	213	20.94 (18.28,23.99)	20.58 (3953.93)	4.36 (4.03)	D	52.00 (10.00,239.50)
Ezetimibe	596	19.82 (18.27,21.51)	19.50 (10,343.1)	4.27 (4.11)	D	56.50 (18.00,239.00)
Flucloxacillin	5	18.46 (7.63,44.67)	18.18 (81.22)	4.18 (1.04)	N	​
Propofol	355	18.01 (16.21,20.00)	17.74 (5571.69)	4.14 (3.92)	D	2.00 (0.00,4.00)
Polymyxin b	5	17.70 (7.32,42.82)	17.44 (77.55)	4.12 (1.03)	N	​
Suxamethonium	34	17.29 (12.32,24.26)	17.04 (513.57)	4.09 (3.05)	N	1.00 (1.00,20.00)
Ritodrine	3	16.92 (5.41,52.90)	16.68 (44.26)	4.06 (0.30)	N	0.00 (0.00,0.00)
alogliptin; metformin	8	16.32 (8.12,32.81)	16.10 (113.42)	4.01 (1.62)	PM	273.00 (245.00,640.00)
Orphenadrine	5	15.17 (6.27,36.65)	14.98 (65.27)	3.90 (0.98)	N	​
Vecuronium	18	15.06 (9.46,23.97)	14.87 (232.99)	3.89 (2.44)	N	1.00 (0.00,13.00)
ciprofloxacin; fluocinolone acetonide	3	14.96 (4.79,46.74)	14.78 (38.57)	3.89 (0.28)	N	2.00 (2.00,2.00)
Norfloxacin	14	14.75 (8.71,25.00)	14.58 (177.14)	3.87 (2.18)	Y	12.00 (2.00,28.00)
Eravacycline	3	14.64 (4.69,45.72)	14.46 (37.62)	3.85 (0.27)	N	​
Fenofibric acid	51	14.39 (10.91,18.96)	14.22 (626.53)	3.83 (3.10)	D	32.50 (3.00,77.00)
Chlortalidone	23	13.54 (8.97,20.43)	13.39 (263.80)	3.74 (2.55)	N	344.00 (344.00,344.00)
Phendimetrazine	3	13.47 (4.31,42.04)	13.32 (34.21)	3.74 (0.25)	N	98.50 (0.00,197.00)
Sevoflurane	103	12.52 (10.31,15.21)	12.40 (1077.95)	3.63 (3.19)	N	0.00 (0.00,1.00)
Danazol	6	12.42 (5.56,27.78)	12.30 (62.33)	3.62 (1.14)	D	3741.00 (3741.00,3741.00)
Levocarnitine	6	12.22 (5.47,27.32)	12.10 (61.14)	3.60 (1.13)	D	35.00 (4.00,40.00)
Isoflurane	16	11.78 (7.20,19.27)	11.66 (156.09)	3.54 (2.14)	N	0.00 (0.00,0.00)
Bromazepam	4	11.54 (4.31,30.90)	11.43 (38.11)	3.51 (0.59)	N	0.00 (0.00,0.00)
Fluphenazine	17	11.54 (7.15,18.61)	11.43 (161.88)	3.51 (2.17)	N	68.00 (5.00,71.00)
Chloroquine	26	10.92 (7.42,16.06)	10.82 (231.82)	3.43 (2.43)	D	1826.00 (1826.00,1826.00)
amoxicillin; clarithromycin; lansoprazole	10	10.85 (5.82,20.23)	10.76 (88.59)	3.43 (1.64)	D	4.00 (2.00,6.00)
Biperiden	8	10.70 (5.33,21.47)	10.61 (69.68)	3.41 (1.39)	N	34.00 (26.00,42.00)
Chlorpromazine	25	10.37 (6.99,15.38)	10.29 (209.66)	3.36 (2.35)	N	5.50 (1.00,10.00)
Sodium polystyrene sulfonate	12	10.32 (5.85,18.22)	10.24 (100.07)	3.36 (1.78)	N	97.00 (19.50,223.50)

CI, confidence interval; IC, information component; IC025, the lower limit of 95% CI, of the IC; PRR, proportional reporting ratio; ROR, reporting odds ratio; χ^2^. chi-squared; Y, yes; N, not.

### 3.3 Top three therapeutic system categories by number of reports

#### 3.3.1 Nernous system drugs

**FIGURE 6:**
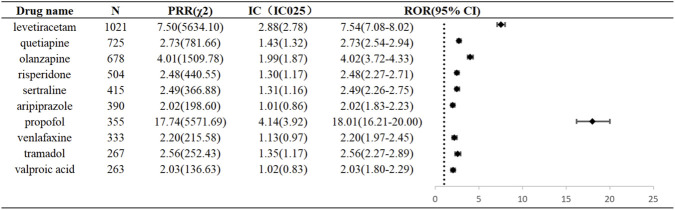
Forest plot of ROR values for the top 10 nervous system drugs for muscle toxicity. Notes: N represents the number of muscular toxicity-related reports for each drug.

Among the 220 drugs with positive signals, 60 were classified as nervous system drugs based on the ATC classification. The top 3 drugs with the highest number of reports were levetiracetam [n = 1021; ROR (95%CI): 7.54 (7.08–8.02)], quetiapine [n = 725; ROR (95%CI): 2.73 (2.54–2.94)] and olanzapine [n = 678; ROR (95%CI): 4.02 (3.72–4.33)]. The 10 most frequently reported nervous system drugs are presented in Figure 6.

#### 3.3.2 Anti-infective drugs

**FIGURE 7:**
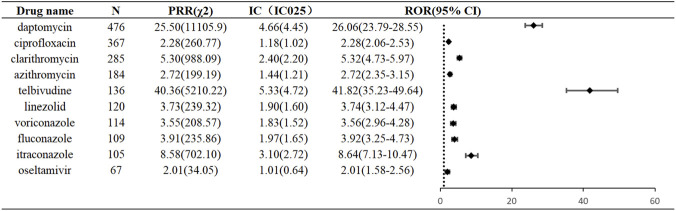
Forest plot of ROR values for the top 10 anti-infective drugs for muscle toxicity.

Among the drugs with positive signals, 49 were classified as anti-infective agents. The most frequently reported agents included daptomycin [n = 476; ROR (95%CI): 26.06 (23.79–28.55)], ciprofloxacin [n = 367; ROR (95%CI): 2.28 (2.06–2.53)], and clarithromycin [n = 285; ROR (95%CI), 5.32 (4.73–5.97)]. The 10 most frequently reported anti-infective drugs are showed in Figure 7.

#### 3.3.3 Cardiovascular drugs

**FIGURE 8:**
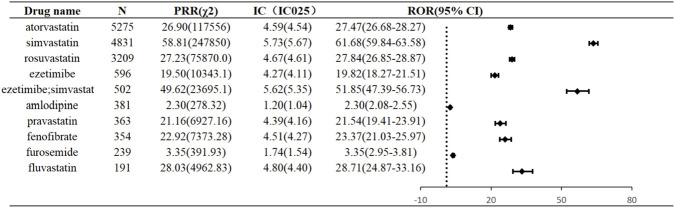
Forest plot of ROR values for the top 10 cardiovascular drugs for muscle toxicity.

A total of 43 cardiovascular drugs showed positive signals. The top 3 drugs with the highest number of reports were atorvastatin [n = 5275; ROR (95%CI): 27.47 (26.68–28.27)], simvastatin [n = 4831; ROR (95%CI): 61.68 (59.84–63.58)], and rosuvastatin [n = 3209; ROR (95%CI), 27.84 (26.85–28.87)]. The 10 most frequently reported cardiovascular drugs are showed in Figure 8.

### 3.4 Top four therapeutic categories in rhabdomyolysis reports

**FIGURE 9:**
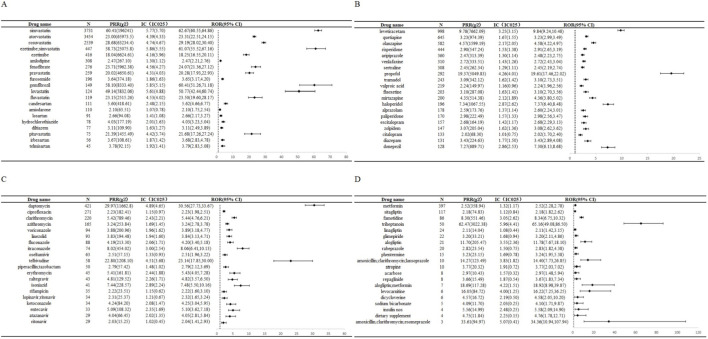
Top 20 drugs by reporting frequency of rhabdomyolysis across four therapeutic categories. Panels **(A–D)** show the top 20 drugs in the cardiovascular, nervous, anti-infective, and digestive and metabolic categories, respectively, ranked by the number of reports.

At the PT level, rhabdomyolysis was the most frequently reported AE. Among cardiovascular drugs, 44 showed positive signals. Simvastatin [n = 3731; ROR (95%CI): 62.67 (60.55–64.86)], atorvastatin [n = 3454; ROR (95%CI): 23.31 (22.51–24.15)], and rosuvastatin [n = 2539; ROR (95%CI): 29.19 (28.02–30.40)] accounted for the highest number of reports. The 20 most frequently reported cardiovascular drugs are showed in Figure 9A.

Within the nervous system category, 65 drugs showed positive signals. The most frequently reported drugs included levetiracetam [n = 998; ROR (95%CI): 9.84 (9.24–10.48)], quetiapine [n = 645; ROR (95%CI): 3.23 (2.99–3.49)], and olanzapine [n = 582; ROR (95%CI): 4.58 (4.22–4.97)]. The 20 most frequently reported nervous drugs are showed in Figure 9B.

In the anti-infective category, 48 drugs demonstrated positive signals, with daptomycin [n = 421; ROR (95%CI): 30.56 (27.73–33.67)], ciprofloxacin [n = 271; ROR (95%CI): 2.23 (1.98–2.51)], and clarithromycin [n = 220; ROR (95%CI): 5.44 (4.76–6.21)] being the most frequently reported. The 20 most frequently reported anti-infective drugs are showed in Figure 9C.

In the digestive and metabolic category, 21 drugs showed positive signals. The most frequently reported drugs were metformin [n = 397; ROR (95%CI): 2.52 (2.28–2.78)], sitagliptin [n = 117; ROR (95%CI): 2.18 (1.82–2.62)], and famotidine [n = 86; ROR (95%CI): 8.34 (6.75–10.32)]. The 20 most frequently reported digestive and metabolic drugs are showed in Figure 9D.

Notably, several drugs identified in this study are not clearly described as being associated with rhabdomyolysis in current product labeling. These include furosemide, amiodarone, hydrochlorothiazide, and diltiazem among cardiovascular drugs; levetiracetam, alprazolam, and diazepam among nervous system drugs; azithromycin and oseltamivir among anti-infective agents; and metformin and sitagliptin among digestive and metabolic drugs. Detailed data are presented in Supplementary Table S7.

### 3.5 DDI analysis for muscle toxicity

DDI signal analysis identified several drug combinations with notable interaction signals for muscle toxicity. Supplementary Figure S2 presents the top 30 drug pairs ranked by reporting frequency. The strongest signals included gemfibrozil-simvastatin [n_111_ = 145; Ω (Ω_025_-Ω_975_): 1.67 (1.43–1.90)], dabrafenib-trametinib [n_111_ = 80; Ω (Ω_025_-Ω_975_): 1.13 (0.82–1.45)], and ezetimibe-rosuvastatin [n_111_ = 73; Ω (Ω_025_-Ω_975_): 0.54 (0.21–0.87)].

### 3.6 Sensitivity analysis

Stratified analyses were performed according to reporter type, sex, and age. Supplementary Table S8 summarizes the results restricted to reports submitted by healthcare professionals. Among the top 50 drugs ranked by reporting frequency, 38 were also identified in the corresponding analysis including all reporters.

**FIGURE 10:**
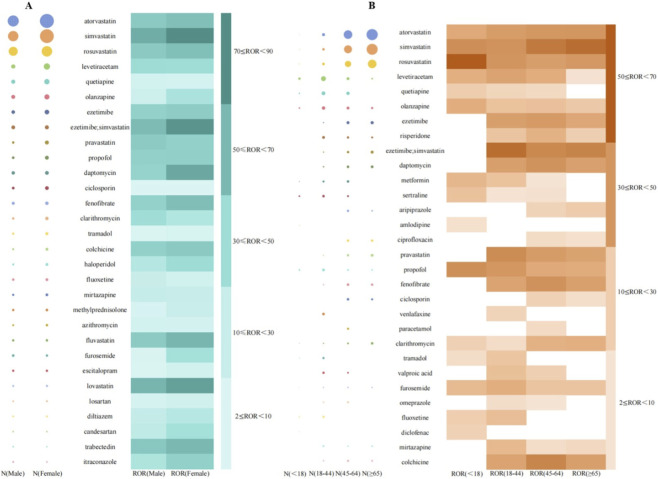
Heatmap of ROR values for muscle toxicity. Panels **(A,B)** show the heatmap of ROR values for muscle toxicity stratified by sex and age. Notes: The accompanying bubble plot (left) illustrates reporting frequency, where bubble size represents the number of reports.

In sex-stratified analyses, simvastatin, the ezetimibe/simvastatin combination, and lovastatin showed higher reporting proportions in female patients compared with male patients (Figure 10A).

In age-stratified analyses, atorvastatin calcium, simvastatin, and rosuvastatin were more frequently reported among older patients (Figure 10B). Detailed results are provided in Supplementary Tables S9, S10.

Supplementary MGPS analysis showed generally consistent signal patterns for the major positive drug-event pairs identified in the primary analyses (Supplementary Table S6).

In sensitivity analyses using minimum report thresholds of ≥10, ≥20, and ≥50 reports, nervous system drugs, anti-infective agents, and cardiovascular drugs consistently remained the three most represented therapeutic categories, which was consistent with the primary analysis. No drugs from the genito-urinary system and sex hormones category met the predefined signal criteria across any of the minimum report thresholds (Supplementary Figure S3).

### 3.7 Time to onset of muscle toxicity-related AEs

TTO could be calculated for 14,476 reports with complete and logically valid date information. The median TTO was 30.00 days (IQR: 4.00–220.00 days), whereas the mean TTO was 324.75 days, with a range of 0–18,113 days. Sensitivity analyses excluding observations above the 99th percentile of the TTO distribution included 14,332 reports. After exclusion of extreme values, the median TTO was 29.00 days and the mean TTO was 272.14 days, with a range of 0–3,930 days. The overall TTO distribution remained generally consistent after exclusion of extreme observations, suggesting that the primary temporal pattern was robust.

### 3.8 Cross-database assessment

A total of 378 drugs related to muscle toxicity were identified from the WHO VigiAccess database. The largest proportion was observed for nervous system drugs (n = 119; 31.48%), followed by cardiovascular drugs (n = 72; 19.05%) and anti-infective drugs (n = 50; 13.23%) (Supplementary Figure S4). Detailed results are presented in Supplementary Tables S11, S12, which showed similar distribution patterns to those observed in the FAERS database.

Forest plots of ROR values for the ten most frequently reported drugs in the nervous, cardiovascular, and anti-infective categories from the WHO VigiAccess database are shown in Supplementary Figure S5-S7. These findings were broadly consistent with the corresponding results from the FAERS database.

## 4 Discussion

Drug-induced muscle toxicity remains an important yet insufficiently characterized safety concern in clinical practice and pharmacovigilance surveillance. In the present study, we performed a large-scale disproportionality analysis of muscle toxicity–related AE reports in the FAERS database from Q1 2004 to Q4 2024. Using multiple disproportionality approaches, including ROR, PRR, and BCPNN analyses, we identified disproportionate reporting signals for 220 drugs potentially associated with muscle toxicity–related events. We further characterized the reporting patterns, clinical features, and therapeutic category distributions of these events, and identified several drugs with high reporting frequencies or notable disproportionality signals. Notably, some detected reporting signals were not prominently described in official product labeling documents at the time of the initial analysis, suggesting potential areas for further pharmacovigilance monitoring and regulatory attention. In addition, generally consistent reporting patterns were observed across different analytical methods and supplementary cross-database assessments. However, these findings should be interpreted cautiously, as disproportionality analyses based on spontaneous reporting systems are inherently subject to reporting bias, underreporting, confounding factors, and incomplete clinical information. Therefore, the identified signals should be regarded as hypothesis-generating reporting associations rather than evidence of confirmed causal relationships.

### 4.1 Baseline characteristics of AE reports

Consistent with previous study, our analysis found a higher reporting proportion of muscle toxicity reports in males compared to females, which is broadly consistent with previous studies that have suggested male sex as a potential risk factor for rhabdomyolysis (Richards et al., 2020). Among the 15 PTs related to muscle toxicity, rhabdomyolysis accounted for the largest number of reports, which supports the focus on this specific AE in subsequent analyses. The number of reports of muscle toxicity showed an overall increasing trend over time, although a slight decrease was observed between 2020 and 2023. Given that muscle toxicity-related events are frequently reported with serious outcomes, such as hospitalization, life-threatening conditions, or death, these findings highlight the clinical relevance of monitoring such events. In the TTO analysis, most reports occurred within the first 30 days following treatment initiation, followed by a gradual decrease over time. This temporal pattern suggests that the early treatment period may represent a critical window for monitoring potential muscle-related adverse reactions. Similar early-onset patterns have also been reported in previous studies evaluating musculoskeletal adverse events associated with statin therapy (Akimoto et al., 2018) and immune checkpoint inhibitors (Touat et al., 2018). Given the markedly skewed distribution of TTO values, median and interquartile range were considered more representative than mean values for describing the overall temporal characteristics of onset.

### 4.2 Key findings across major pharmacological classes and mechanistic insights

Based on both reporting frequency and disproportionality strength, cardiovascular, nervous system, anti-infective, and digestive and metabolic agents represented the major therapeutic categories associated with muscle toxicity–related reporting signals in the present study. Below, we contextualize these findings in relation to existing literature and discuss potential mechanisms that may contribute to the observed reporting patterns.

#### 4.2.1 Cardiovascular drugs

Among cardiovascular drugs, statins demonstrated some of the strongest and most consistent reporting signals for muscle toxicity and rhabdomyolysis in the present study. As the cornerstone of lipid-lowering therapy, statins are well recognized for their association with a spectrum of muscle-related AEs ranging from mild myalgia to severe rhabdomyolysis (Stroes et al., 2015). Previous studies have suggested that mitochondrial dysfunction, impaired cellular energy metabolism, and reduced coenzyme Q10 synthesis secondary to HMG-CoA reductase inhibition may contribute to statin-associated muscle injury (Vaklavas et al., 2009; Brown et al., 2014). Consistent with previous FAERS- and VigiBase-based pharmacovigilance studies, significant disproportionality signals were observed for multiple statins in our analysis, particularly simvastatin, lovastatin, rosuvastatin, and atorvastatin, supporting the established association between statin exposure and muscle toxicity–related AEs.

Beyond statins, notable reporting signals were also identified for several commonly prescribed antihypertensive agents, particularly calcium channel blockers (CCBs) and diuretics. Amlodipine demonstrated a disproportionality signal for muscle toxicity–related events. Although amlodipine is generally considered to have a favorable safety profile, it may weakly inhibit cytochrome P450 3A4 (CYP3A4), thereby increasing plasma concentrations of concomitantly administered statins and potentially increasing the risk of statin-associated myopathy (Schröder et al., 2016; Banakh et al., 2017; Khan et al., 2018). Similarly, diltiazem has been reported to increase systemic exposure to CYP3A4-metabolized statins, which may contribute to elevated rhabdomyolysis risk in combination therapy settings (Fuah and Lim, 2021). Consistent with this possibility, our DDI analysis identified a positive interaction signal between diltiazem and simvastatin (Ω = 1.82). Experimental studies have additionally suggested that certain CCBs may influence intracellular calcium homeostasis in skeletal muscle cells, although the clinical relevance of these findings remains uncertain (Subra et al., 2012). Therefore, the observed signals for CCBs should be interpreted cautiously, as they may partly reflect DDIs, polypharmacy, or underlying comorbidities rather than direct myotoxic effects.

Diuretics, including furosemide and hydrochlorothiazide, also demonstrated disproportionate reporting signals in the present study. Current evidence suggests that these associations may be indirectly related to electrolyte disturbances, particularly hypokalemia and hypophosphatemia, rather than direct muscle toxicity (Ruisz et al., 2013; Sawada et al., 2020; Thillard et al., 2024). Severe potassium depletion may impair Na^+^/K^+^-ATPase activity, promote intracellular calcium overload, and disrupt skeletal muscle energy metabolism, potentially contributing to rhabdomyolysis. Similarly, severe hypophosphatemia has been associated with muscle cell injury and rhabdomyolysis in susceptible patients. Given that diuretics are frequently prescribed in elderly patients and individuals with cardiovascular or renal comorbidities, confounding factors such as dehydration, renal dysfunction, and concomitant medications should also be considered when interpreting these signals.

Amiodarone also demonstrated a disproportionate reporting signal in our analysis. Although amiodarone-associated muscle toxicity appears to be uncommon, previous case reports have described necrotizing myopathy and marked creatine kinase elevation during treatment (Carella et al., 1987; Clouston and Donnelly, 1989; Flanagan et al., 2012). Potential mechanisms may involve drug accumulation within muscle tissue and subsequent myocellular injury, although available evidence remains limited. Notably, postmarketing reports of rhabdomyolysis have also been described in official product labeling documents.

Overall, the findings related to cardiovascular drugs highlight the potential contribution of polypharmacy and pharmacokinetic DDIs to muscle toxicity–related reporting signals. Given the widespread use of combination cardiovascular therapies in clinical practice, careful monitoring of high-risk drug combinations and patient-specific risk factors may be warranted.

#### 4.2.2 Nervous system drugs

Nervous system drugs represented one of the major therapeutic categories associated with disproportionate reporting signals for muscle toxicity and rhabdomyolysis in the present study. Compared with cardiovascular drugs, where pharmacokinetic interactions are major contributors, muscle toxicity associated with psychotropic and antiepileptic agents appears to involve more complex and multifactorial mechanisms, including central nervous system depression, prolonged immobilization, neuropsychiatric complications, seizures, hyperthermia, and concomitant medications. These factors may substantially complicate causal interpretation in spontaneous reporting systems.

Among atypical antipsychotics, quetiapine, olanzapine, risperidone, and aripiprazole all demonstrated notable disproportionality signals, consistent with previous pharmacovigilance studies linking these agents to rhabdomyolysis (Yin et al., 2025). Quetiapine has been most extensively described in the literature and is specifically mentioned in product labeling as a potential cause of rhabdomyolysis, particularly in overdose settings. Several mechanisms have been proposed, including dopamine blockade–related muscle rigidity, neuroleptic malignant syndrome (NMS), agitation-associated muscle injury, and prolonged immobilization during altered mental status (Poyurovsky and Weizman, 2023). In addition, serotonergic and dopaminergic dysregulation may increase skeletal muscle membrane permeability and contribute to creatine kinase elevation (Yin et al., 2025). Quetiapine has also been prescribed off-label for fibromyalgia and chronic musculoskeletal pain, potentially reflecting its effects on sleep regulation, mood, and pain perception pathways (Velasco-Montes et al., 2012; Calandre and Rico-Villademoros, 2012; Li et al., 2020). However, many reported cases involved additional risk factors, including dehydration, infection, strenuous activity, organ dysfunction, or concomitant myotoxic medications, making direct causal attribution difficult. Therefore, the signals identified in this study should be interpreted as hypothesis-generating reporting associations rather than confirmed drug-induced myotoxicity.

Antiepileptic drugs also demonstrated relevant reporting signals in our analysis, particularly levetiracetam and valproic acid. Previous studies and case reports have described rhabdomyolysis following levetiracetam exposure (Jiang et al., 2016; Al-Anbagi et al., 2025), whereas evidence for valproic acid remains limited. Interpretation of these signals is complicated by the fact that seizures themselves are a recognized cause of rhabdomyolysis. Sustained tonic-clonic muscle activity may induce hypermetabolism, intracellular calcium overload, ATP depletion, and subsequent skeletal muscle injury. In addition, mitochondrial dysfunction and metabolic disturbances associated with antiepileptic therapy have been proposed as potential contributing mechanisms. Our findings therefore expand existing pharmacovigilance observations and support continued monitoring of muscle-related adverse events during long-term antiepileptic treatment, particularly in patients with recurrent seizures or polypharmacy.

Signals were also identified for benzodiazepines and sedative-hypnotic agents, including alprazolam, diazepam, and eszopiclone, as well as the selective serotonin reuptake inhibitor escitalopram. Unlike statin-associated muscle toxicity, rhabdomyolysis associated with these agents may often occur secondary to prolonged unconsciousness, immobilization, overdose, substance misuse, or serotonin-related toxicity rather than direct skeletal muscle injury. FDA safety communications have previously highlighted serious adverse events associated with diazepam, including rhabdomyolysis in susceptible individuals (He et al., 2024). Similarly, a case report described rhabdomyolysis and femoral artery microthrombosis following intravenous alprazolam injection in a drug-dependent patient (Wang and Chew., 2015), although the complex clinical setting limited causal interpretation. Previous pharmacovigilance analyses have also suggested potential associations between SSRIs, including escitalopram, and rhabdomyolysis (Ohyama et al., 2025), which is consistent with our findings. One plausible explanation involves serotonin syndrome–related hyperthermia and neuromuscular hyperactivity, both of which may contribute to skeletal muscle breakdown. In contrast, direct clinical evidence linking eszopiclone to rhabdomyolysis remains sparse, and the signal observed in this study should therefore be interpreted cautiously.

Collectively, the findings for nervous system drugs highlight the multifactorial and clinically heterogeneous nature of muscle toxicity in neuropsychiatric populations. In many cases, the observed reporting signals may reflect interactions among drug exposure, underlying neurologic or psychiatric disorders, behavioral risk factors, overdose, immobilization, and concomitant medications. From a clinical perspective, increased vigilance may be warranted in high-risk patients, particularly those receiving multiple psychotropic agents or presenting with seizures, altered consciousness, or prolonged immobilization. Continued pharmacovigilance surveillance and further pharmacoepidemiologic studies are needed to clarify the clinical significance and biological mechanisms underlying these reporting signals.

#### 4.2.3 Anti-infective drugs

Among anti-infective agents, several drugs identified in our analysis are not prominently labeled for muscle toxicity or rhabdomyolysis in current prescribing information, including azithromycin, voriconazole, and oseltamivir. These findings therefore warrant careful interpretation in the context of underlying infection, polypharmacy, and patient-related risk factors.

Azithromycin, one of the most widely prescribed macrolide antibiotics, demonstrated a disproportional reporting signal for rhabdomyolysis in our study. Existing evidence, however, remains limited largely to isolated case reports (Bouquié et al., 2011; Finsterer et al., 2022; Kato et al., 2016). Importantly, many reported patients had concurrent predisposing conditions, including fever, strenuous physical activity, systemic infection, or concomitant use of other potentially myotoxic drugs. These factors substantially complicate causal attribution. In addition, macrolides may contribute indirectly to muscle toxicity through pharmacokinetic interactions involving CYP3A4 inhibition, thereby increasing exposure to co-administered drugs associated with myopathy. Consequently, the signal observed in this study should be interpreted as a potential reporting association rather than evidence of a direct myotoxic effect of azithromycin itself. From a clinical perspective, awareness may be particularly relevant in patients with multiple concurrent risk factors or complex medication regimens rather than during routine short-term azithromycin use.

Voriconazole also showed a notable disproportionality signal, consistent with previous pharmacovigilance analyses suggesting rhabdomyolysis as a potential emerging adverse event associated with triazole antifungals (Tian et al., 2025). Although only a limited number of clinical cases have been described (Li et al., 2017; Alawfi et al., 2021), several mechanistic hypotheses may explain this association. Voriconazole is primarily metabolized through CYP2C19, CYP2C9, and CYP3A4 pathways, with CYP2C19 serving as the major metabolic enzyme (Wang et al., 2009). Because CYP2C19 exhibits substantial genetic polymorphism, poor metabolizers may experience markedly elevated plasma voriconazole concentrations, thereby increasing susceptibility to concentration-dependent toxicities. Moreover, concomitant administration of CYP inhibitors may further enhance systemic exposure. These pharmacokinetic characteristics may partially explain the variability in reported muscle-related AEs. The consistency between our findings and previous reports suggests that therapeutic drug monitoring and careful evaluation of drug-drug interactions may be particularly important during prolonged voriconazole therapy.

Within antiviral agents, oseltamivir generated a detectable reporting signal; however, interpretation remains challenging because influenza infection itself is a recognized cause of rhabdomyolysis. Influenza A virus has been reported to directly invade skeletal muscle tissue and induce muscle fiber necrosis, potentially leading to acute kidney injury (Puttagunta et al., 2018). Therefore, distinguishing disease-related muscle injury from drug-associated toxicity is inherently difficult in this setting. Current evidence linking oseltamivir itself to rhabdomyolysis remains sparse, with only isolated case reports available. Accordingly, the present findings should be regarded as hypothesis-generating observations requiring further validation rather than confirmation of a causal relationship.

Signals were also identified for the nucleos(t)ide analogues telbivudine and entecavir, both of which are widely used for long-term treatment of chronic hepatitis B infection. Previous studies have mainly described mild muscle-related symptoms, including myalgia and weakness, particularly during prolonged exposure (Caroleo et al., 2011; Wang et al., 2022). Telbivudine-associated myopathy has been linked to mitochondrial dysfunction and impaired oxidative metabolism (Fontana, 2009), although experimental findings have been inconsistent (Standring et al., 2001). Genetic susceptibility may also contribute, as variants in the RRM2B gene have been associated with telbivudine-related myopathy (Hernández-Laín et al., 2015). In contrast, mechanistic evidence for entecavir-related muscle toxicity remains extremely limited. Given the chronic nature of antiviral therapy and the frequent coexistence of hepatic dysfunction, metabolic abnormalities, and concomitant medications, clinicians should remain attentive to unexplained muscle symptoms or creatine kinase elevations during long-term treatment, particularly in vulnerable patients.

Overall, the anti-infective drug class illustrates the complexity of interpreting pharmacovigilance signals for muscle toxicity. In many cases, infection severity, systemic inflammation, organ dysfunction, and multidrug exposure may substantially contribute to the observed associations. These findings therefore underscore the importance of integrating pharmacovigilance signals with clinical context when evaluating potential drug-related muscle injury.

#### 4.2.4 Digestive and metabolic system drugs

Signals related to muscle toxicity were also observed for several glucose-lowering agents and proton pump inhibitors (PPIs). Because these drugs are commonly prescribed for long-term use, even uncommon muscle-related adverse events may be clinically relevant in susceptible patients.

Metformin showed a disproportionality signal for rhabdomyolysis in the present analysis. At the time of the initial data extraction, rhabdomyolysis was not specifically described in the official prescribing information for metformin. During manuscript revision, however, updated labeling documents incorporated rhabdomyolysis into post-marketing adverse reaction reports. Although spontaneous reporting data cannot establish causality, several clinical reports have described rhabdomyolysis in the setting of metformin overdose or severe metabolic derangements associated with metformin accumulation (Sánchez-Rubio Ferrández et al., 2013). Experimental evidence has also suggested that high-dose metformin may aggravate rhabdomyolysis-associated acute kidney injury through mitochondrial dysfunction and metabolic stress (Cai et al., 2023). These observations suggest that muscle toxicity associated with metformin may occur primarily under specific high-risk conditions, particularly in patients with renal impairment or severe systemic illness.

Sitagliptin, a dipeptidyl peptidase-4 (DPP-4) inhibitor, also demonstrated a positive reporting signal. Published case reports have mainly involved concomitant exposure to sitagliptin and statins (Atapour et al., 2024; Patel et al., 2019), making it difficult to distinguish the independent contribution of sitagliptin from potential DDIs. A recent pharmacovigilance study evaluating musculoskeletal adverse events associated with DPP-4 inhibitors similarly identified signals for rhabdomyolysis involving sitagliptin, linagliptin, and alogliptin (Guo et al., 2026). At present, however, mechanistic evidence remains limited, and the clinical significance of these signals requires further clarification.

Among gastrointestinal agents, rabeprazole and several other PPIs were associated with reporting signals for rhabdomyolysis. Current evidence remains inconclusive. A review of published case reports did not support a clear causal association between PPI exposure and rhabdomyolysis (Colmenares and Pappas, 2017), whereas analyses based on large spontaneous reporting databases, including FAERS, have detected disproportionate reporting signals (Duncan and Howden, 2017). In many reported cases, concomitant statin therapy or multiple comorbidities were present, suggesting that polypharmacy and underlying clinical conditions may have contributed to the observed associations. Similar findings were reported in a recent pharmacovigilance analysis of PPIs (Sun et al., 2024), supporting the reproducibility of the signal across datasets. In clinical practice, these findings may warrant additional attention in patients receiving combination therapy or in those with pre-existing risk factors for muscle injury.

## 5 Limitations

This study provides a comprehensive overview of muscle toxicity–related reports based on the FAERS database; however, several limitations should be considered when interpreting the findings. Firstly, the spontaneous nature of FAERS reporting introduces inherent bias. Not all reports originate from healthcare professionals, and underreporting, incomplete data, or inaccuracies in event attribution may affect data quality. Secondly, although disproportionality analysis methods are widely used in pharmacovigilance, they are susceptible to confounding factors, including comorbidities, polypharmacy, and differences in drug utilization patterns across populations. These factors may influence signal detection and interpretation. Thirdly, this study is observational and exploratory. While signals for muscle toxicity were identified for multiple drugs, causal relationships cannot be established, and further studies are needed to evaluate these findings. To improve the robustness of the analysis, stratified analyses were conducted according to sex, age, and reporter type. Consistent reporting patterns observed across these subgroups provide supportive evidence for the stability of the identified signals. External validation using the WHO VigiAccess database showed broadly similar distribution patterns, which may further support the reliability of the findings. In addition, supplementary analysis using the MGPS method showed generally consistent signal patterns with the primary analyses. Nevertheless, certain limitations remain. Analyses of potential drug–drug interactions were not performed due to data constraints, although such interactions may play an important role in the occurrence of muscle toxicity. Despite these limitations, the findings provide an overview of reporting patterns for muscle toxicity and may support signal detection and hypothesis generation for future research.

## 6 Conclusion

This study provides a comprehensive pharmacovigilance evaluation of muscle toxicity–related AE reports and the corresponding drugs using FAERS data. By integrating multiple disproportionality analyses, stratified analyses, and supplementary cross-database consistency assessments, we identified several drugs with notable reporting frequencies and disproportionality signals. Some drugs, including metformin and eszopiclone, showed disproportionate reporting signals that were not prominently reflected in earlier versions of official labeling documents at the time of the initial analysis. During manuscript revision, several official labeling documents were updated to include additional information related to muscle toxicity. However, these findings should be interpreted cautiously, as disproportionality analyses cannot establish causal relationships and may be influenced by confounding factors and reporting biases inherent to spontaneous reporting systems. Further clinical and pharmacoepidemiologic studies are required to clarify the clinical relevance of these findings. Overall, the present study is intended to provide hypothesis-generating evidence and support continued pharmacovigilance monitoring of potential drug-associated muscle toxicity.

## Data Availability

The datasets presented in this study can be found in online repositories. The names of the repository/repositories and accession number(s) can be found in the article/Supplementary Material.

## Glossary

AE: Adverse event
FAERS: the U.S. Food and Drug Administration Adverse Event Reporting System
PT: Preferred Term
PS: Primary Suspect
ROR: Reporting Odds Ratio
aROR: Adjusted reporting Odds Ratio
PRR: Proportional Reporting Ratio
BCPNN: Bayesian Confidence Propagation Neural Network
ADR: Adverse drug reaction
NSAIDs: Nonsteroidal anti-inflammatory drugs
SAMS: Statin-associated muscle symptoms
SOCs: System Organ Classes
SMQ: Standardized MedDRA Queries
MedDRA: Medical Dictionary for Regulatory Activities
IC: Information component
MGPS: Multi-item Gamma Poisson shrinker
TTO: Time to onset
IQR: Interquartile ranges
RCTs: Randomized clinical trials
CASQ: Calsequestrin
AKI: Acute kidney injury
ARBs: Angiotensin II receptor blockers
CYP3A4: cytochrome P450 3A4
BZDs: benzodiazepines
SSRI: selective serotonin reuptake inhibitor
DPP-4: dipeptidyl peptidase-4
GLP-1: glucagon-like peptide-1
GIP: glucose-dependent insulinotropic peptide
PPIs: proton-pump inhibitors
MDV: Medical Data Vision
