# Supplementary material for: Muscle toxicity reports in FAERS: a disproportionality analysis with focus on rhabdomyolysis and cross-database assessment
Source: Front Pharmacol. 2026 Jun 22;17:1852621. doi: 10.3389/fphar.2026.1852621 (PMC13334127; doi:10.3389/fphar.2026.1852621)
Supplement: Supplementary file 1 [file Supplementaryfile1.docx]

# Supplementary materials

1. **List of Preferred Terms (PTs)**

**Table S1.** Preferred Terms (PTs) included in the narrow SMQ “rhabdomyolysis/myopathy” and their inclusion status in downstream analyses

| **PT code** | **PT name** | **Included in downstream analyses** |
| --- | --- | --- |
| 10028631 | Myoglobin urine present | Yes |
| 10059888 | Myoglobin blood present | Yes |
| 10028625 | Myoglobin blood increased | Yes |
| 10039020 | Rhabdomyolysis | Yes |
| 10074769 | Necrotising myositis | Yes |
| 10028641 | Myopathy | Yes |
| 10058735 | Myoglobinaemia | Yes |
| 10086278 | Muscle infarction | No |
| 10028320 | Muscle necrosis | Yes |
| 10081524 | Thyrotoxic myopathy | No |
| 10090562 | Hypothyroid myopathy | No |
| 10088386 | Exertional rhabdomyolysis | Yes |
| 10087983 | Diabetic myonecrosis | No |
| 10028648 | Myopathy toxic | Yes |
| 10028629 | Myoglobinuria | Yes |

Notes: Four PTs (muscle infarction, thyrotoxic myopathy, hypothyroid myopathy, and diabetic myonecrosis) were excluded from downstream analyses because their underlying etiologies are more commonly related to endocrine, metabolic, or ischemic disorders rather than drug-induced muscle toxicity, which could introduce substantial clinical heterogeneity into signal detection analyses.

1. **Methods**

Disproportionation analysis is a data mining method, which is mainly used to evaluate the correlation between drugs and adverse reactions. The core principle is to use a 2 × 2 contingency table to compare the frequency of adverse events observed in the exposed group and the non-exposed group, so as to quantify the association between drugs and adverse events (AEs). When the proportion of AEs in the exposed group exceeded that in the unexposed group, it was inferred that there was an association between drugs and specific AEs, indicating the presence of a disproportionation signal. After exceeding the threshold, the larger the signal value, the stronger the signal. In this study, we used four disproportional analysis methods: Reporting odds ratio (ROR), proportional reporting ratio (PRR), and bayesian confidence propagation neural network (BCPNN).

Specific data analysis methods are listed below:

**Table S2.** Two-by-two contingency table for disproportionality analysis

| Item | Target adverse events reported | Other adverse events reported |  | Total |
| --- | --- | --- | --- | --- |
| Target drugs | a | b |  | a + b |
| Other drugs | c | d |  | c + d |
| Total | a + c | b + d |  | a + b + c + d |

**Table S3.** The principles of disproportionate measurement and the criteria for signal detection

| Method | Calculation formula | ﻿Criteria |
| --- | --- | --- |
| ROR | $ROR=\frac{a / c}{b / d}$ | a ≥ 3  ROR≥1  95%CI (lower limit) > 1 |
|  | $SE(lnROR)=\sqrt{\frac{1}{a}+\frac{1}{b}+\frac{1}{c}+\frac{1}{d}}$ |  |
|  | $95\%CI= e^{\ln\left( ROR \right)\pm1.96se}$ |  |
| PRR | $PRR=\frac{a / (a+b)}{c / (c+d)}$ | a ≥ 3  PRR ≥ 2  $\chi2\geq4$  95%CI (lower limit) > 1 |
|  | $SE(lnPRR)=\sqrt{\frac{1}{a}-\frac{1}{a+b}+\frac{1}{c}-\frac{1}{c+d}}$ |  |
|  | $95\%CI= e^{\ln\left( PRR \right)\pm1.96se}$ |  |
|  | $\chi2 =\frac{{(ad-bc)}^{2}(a+b+c+d)}{( a+b)(a+c)(c+d)(b+d)}$ |  |
| BCPNN | IC=${log}_{2}\frac{p(x,y)}{p(x)p(y)}={log}_{2}\frac{a(a+b+c+d)}{(a+b)(a+c)}$ | IC025>0 |
|  | E(IC)=${log}_{2}\frac{(a+\gamma11)(a+b+c+d+\alpha)(a+b+c+d+\beta)}{（a+b+c+d+\gamma）(a+b+\alpha1)(a+c+\beta1)}$ |  |
|  | $V\left( IC \right)=\frac{1}{{(ln2)}^{2}}\{\left[ \frac{\left( a+b+c+d \right)-a+\gamma-\gamma11}{\left( a+\gamma11 \right)\left( 1+a+b+c+d+\gamma\right)} \right]+\left[ \frac{\left( a+b+c+d \right)-\left( a+b \right)+\alpha-\alpha1}{\left( a+b+\alpha1 \right)\left( 1+a+b+c+d+\alpha\right)} \right]+\left[ \frac{\left( a+b+c+d \right)-\left( a+c \right)+\beta-\beta1}{\left( a+c+\beta1 \right)\left( 1+a+b+c+d+\beta\right)} \right]\}$ |  |
|  | $\gamma=\gamma11\frac{(a+b+c+d+\alpha)(a+b+c+d+\beta)}{(a+b+\alpha1)(a+c+\beta1)}$ |  |
|  | IC025=IC-2SD=E(IC)-2$\sqrt{V(IC)}$  $\alpha1=\beta1=1；\alpha=\beta=2；\gamma11=1$ |  |
| MGPS | $EBGM=\frac{a(a+b+c+d)}{\left( a+c \right)(a+b)}$ | EBGM05>2 |
|  | $SE(lnEBGM)=\sqrt{\frac{1}{a}+\frac{1}{b}+\frac{1}{c}+\frac{1}{d}}$ |  |
|  | $95\%CI= e^{\ln\left( EBGM \right)\pm1.96se}$  $EBGM05= e^{\ln\left( EBGM \right)-1.96se}$ |  |

Equation: a, number of reports containing both the target drug and target adverse event; b, number of reports containing other adverse event of the target drug; c, number of reports containing the target adverse event of other drugs; d, number of reports containing other drugs and other adverse events. ROR, reporting odds ratio; CI, confidence interval; PRR, proportional reporting ratio; χ2, chi-squared; BCPNN, bayesian confidence propagation neural network; IC, information component; IC025, the lower limit of 95%CI of the IC; 95%CI, 95% confidence interval; χ2, chi-squared; E (IC), the IC expectations; V (IC), the variance of IC; MGPS: Multi-item Gamma Poisson Shrinker , EBGM: empirical bayesian geometric mean, EBGM05: lower limit of 95% CI of EBGM.

Although the degree of drug suspicion was defined as the primary suspicion during mining, the interference of other confounding factors, such as the influence of drug combination, could not be excluded. Therefore, in this study, in addition to data analysis by conventional ratio imbalance method, Ω contraction measurement method was also used to mine drug combination interactions and screen out drug-drug combinations that increase the risk of target AE.

Both the frequency method and Bayesian method can be used to estimate the Ω contraction measurement, and the signal detection threshold is Ω025 > 0, the lower limit of 95% confidence interval of the Ω contraction measurement.

Table S4. Combined drug signal detection 4 × 2 contingency table.

| Drug combination | Target AE | Other AEs | Total |
| --- | --- | --- | --- |
| Both drugs A and B were exposed | *n*_111_ | *n*_110_ | *n*_11+_ |
| Only drug A was exposed | *n*_101_ | *n*_100_ | *n*_10+_ |
| Only drug B was exposed | *n*_011_ | *n*_010_ | *n*_01+_ |
| Drugs A and B were not exposed | *n*_001_ | *n*_000_ | *n*_00+_ |
| Total | *n_++_*_1_ | *n*_++0_ | *n*_+++_ |

The incidence of target AE under different drug exposure conditions were:

$f_{11}=\frac{n_{111}}{n_{11+}}{; f}_{10}=\frac{n_{101}}{n_{10+}}$; $f_{01}=\frac{n_{011}}{n_{01+}}$; $f_{00}=\frac{n_{001}}{n_{00+}}$

$$g_{11}= 1 -\frac{1}{\max\left( \frac{f_{00}}{1-f_{00}},\frac{f_{10}}{1-f_{10}} \right)+\max\left( \frac{f_{00}}{1-f_{00}},\frac{f_{01}}{1-f_{01}} \right)-\frac{f_{00}}{1-f_{00}}+ 1}$$

E_111_=g_11_×n_11+_

$\Omega={log}_{2}\frac{n_{111}+0.5}{E_{111}+0.5}$，$\Omega_{025}=\Omega-1.96\sqrt{\log_{2} \frac{n_{111}+0.5}{E_{111}+0.5}}$

*n111*, reported number of adverse events targeted by combinations of two drugs; *E111*, expected value of adverse events targeted by combinations of two drugs; *Ω_025_*, lower limit of the 95% CI of Ω.

1. **Others**

**Table S5.** Number and proportion of AE reports from reporting country

| **Reporting countries** | **Cases, n (%)** |
| --- | --- |
| United States of America | 14179(30.01) |
| France | 4665( 9.87) |
| Japan | 4426( 9.37) |
| Not Specified | 3620( 7.66) |
| United Kiongdom | 3444( 7.29) |
| Germany | 2538( 5.37) |
| Italy | 2006( 4.25) |
| Canada | 1741( 3.69) |
| Spain | 1304( 2.76) |
| Australia | 984( 2.08) |
| China | 875( 1.85) |
| Netherlands | 581( 1.23) |
| Sweden | 567( 1.20) |
| Switzerland | 553( 1.17) |
| Portugal | 463( 0.98) |
| Turkey | 392( 0.83) |
| India | 364( 0.77) |
| Denmark | 328( 0.69) |
| Belgium | 307( 0.65) |
| Greece | 290( 0.61) |
| Finland | 275( 0.58) |
| Poland | 252( 0.53) |
| Taiwan | 224( 0.47) |
| Norway | 205( 0.43) |
| Austria | 197( 0.42) |
| Korea | 189( 0.40) |
| Croatia | 173( 0.37) |
| Czech Republic | 169( 0.36) |
| Brazil | 160( 0.34) |
| Ireland | 159( 0.34) |
| Iran | 118( 0.25) |
| Israel | 103( 0.22) |
| New Zealand | 84( 0.18) |
| Romania | 81( 0.17) |
| Hongkong | 75( 0.16) |
| Hungary | 67( 0.14) |
| Russia | 64( 0.14) |
| Singapore | 64( 0.14) |
| Slovenia | 63( 0.13) |
| Colombia | 58( 0.12) |
| Malaysia | 54( 0.11) |
| Thailand | 53( 0.11) |
| Slovakia | 48( 0.10) |
| Mexico | 42( 0.09) |
| South Africa | 42( 0.09) |
| Tunisia | 41( 0.09) |
| Saudi Arabia | 40( 0.08) |
| Sri Lanka | 38( 0.08) |
| United Arab Emirates | 31( 0.07) |
| Philippines | 29( 0.06) |
| Morocco | 28( 0.06) |
| Argentina | 27( 0.06) |
| Eritrea | 24( 0.05) |
| Serbia | 24( 0.05) |
| Qatar | 23( 0.05) |
| Kuwait | 23( 0.05) |
| Chile | 22( 0.05) |
| Puerto Rico | 20( 0.04) |
| Luxembourg | 19( 0.04) |
| Bahrain | 17( 0.04) |
| Bulgaria | 12( 0.03) |
| Vietnam | 12( 0.03) |
| Pakistan | 11( 0.02) |
| Bangladesh | 10( 0.02) |
| Nepal | 9( 0.02) |
| Egypt | 8( 0.02) |
| Latvia | 8( 0.02) |
| Lebanon | 8( 0.02) |
| Venezuela | 8( 0.02) |
| Indonesia | 8( 0.02) |
| Malta | 7( 0.01) |
| Peru | 7( 0.01) |
| Faroe Islands | 6( 0.01) |
| Lithuania | 5( 0.01) |
| Jordan | 5( 0.01) |
| North Korea | 4( 0.01) |
| Dominica Rep. | 4( 0.01) |
| Cyprus | 4( 0.01) |
| Anguilla | 3( 0.01) |
| Costa Rica | 3( 0.01) |
| Nigeria | 3( 0.01) |
| Swaziland | 3( 0.01) |
| Guatemala | 3( 0.01) |
| Uruguay | 3( 0.01) |
| Syria | 3( 0.01) |
| Benin | 2( 0.00) |
| Georgia | 2( 0.00) |
| Cameroon | 2( 0.00) |
| Cayman Islands | 2( 0.00) |
| Sierra Leone | 2( 0.00) |
| Ukraine | 2( 0.00) |
| Algeria | 1( 0.00) |
| Afghanistan | 1( 0.00) |
| Oman | 1( 0.00) |
| Estonia | 1( 0.00) |
| Paraguay | 1( 0.00) |
| Panama | 1( 0.00) |
| Iceland | 1( 0.00) |
| Burkina-faso | 1( 0.00) |
| Honduras | 1( 0.00) |
| Ghana | 1( 0.00) |
| Kenya | 1( 0.00) |
| Réunion | 1( 0.00) |
| United States Minor Outlying Islands | 1( 0.00) |
| European Union | 1( 0.00) |
| EI Salvador | 1( 0.00) |
| Senegal | 1( 0.00) |
| Trinidad and Tobago | 1( 0.00) |
| Brunei | 1( 0.00) |
| New Caledonia | 1( 0.00) |
| Jamaica | 1( 0.00) |

Notes: n represents the case-level, which is the number of patientss associated with muscle toxicity. The denominator for the percentage is 47,241 patients.

**Table S6.** 220 drugs with positive signals for muscle toxicity in the FAERS database

| **Drug name** | **ATC(Code)** | **Case reports** | **ROR(95%CI)** | **PRR(χ²)** | **IC(IC025)** | **EBGM(EBGM_05_)** |
| --- | --- | --- | --- | --- | --- | --- |
| atorvastatin | C10AA | 5275 | 27.47(26.68,28.27) | 26.90(117556) | 4.59(4.54) | 24.13(23.44) |
| simvastatin | C10AA | 4831 | 61.68(59.84,63.58) | 58.81(247850) | 5.73(5.67) | 53.15(51.56) |
| rosuvastatin | C10AA | 3209 | 27.84(26.85,28.87) | 27.23(75870.0) | 4.67(4.61) | 25.52(24.62) |
| levetiracetam | N03AX | 1021 | 7.54(7.08,8.02) | 7.50(5634.10) | 2.88(2.78) | 7.36(6.92) |
| quetiapine | N05AH | 725 | 2.73(2.54,2.94) | 2.73(781.66) | 1.43(1.32) | 2.70(2.51) |
| olanzapine | N05AH | 678 | 4.02(3.72,4.33) | 4.01(1509.78) | 1.99(1.87) | 3.96(3.67) |
| ezetimibe | C10AX | 596 | 19.82(18.27,21.51) | 19.50(10343.1) | 4.27(4.11) | 19.28(17.77) |
| risperidone | N05AX | 504 | 2.48(2.27,2.71) | 2.48(440.55) | 1.30(1.17) | 2.46(2.26) |
| ezetimibe;simvastatin | C10BA | 502 | 51.85(47.39,56.73) | 49.62(23695.1) | 5.62(5.35) | 49.13(44.91) |
| daptomycin | J01XX | 476 | 26.06(23.79,28.55) | 25.50(11105.9) | 4.66(4.45) | 25.26(23.06) |
| metformin | A10BA | 466 | 2.23(2.03,2.44) | 2.22(310.99) | 1.15(1.01) | 2.21(2.02) |
| sertraline | N05BX;N06AB | 415 | 2.49(2.26,2.75) | 2.49(366.88) | 1.31(1.16) | 2.48(2.25) |
| aripiprazole | N05AX | 390 | 2.02(1.83,2.23) | 2.02(198.60) | 1.01(0.86) | 2.01(1.82) |
| amlodipine | C08CA | 381 | 2.30(2.08,2.55) | 2.30(278.32) | 1.20(1.04) | 2.29(2.07) |
| ciprofloxacin | D06AX;G01AA;J01MA;S01AE;S02AA;S03AA | 367 | 2.28(2.06,2.53) | 2.28(260.77) | 1.18(1.02) | 2.27(2.04) |
| pravastatin | C10AA | 363 | 21.54(19.41,23.91) | 21.16(6927.16) | 4.39(4.16) | 21.01(18.93) |
| propofol | N01AX | 355 | 18.01(16.21,20.00) | 17.74(5571.69) | 4.14(3.92) | 17.62(15.86) |
| fenofibrate | C10AB | 354 | 23.37(21.03,25.97) | 22.92(7373.28) | 4.51(4.27) | 22.76(20.48) |
| ciclosporin | L04AD;S01XA | 343 | 2.12(1.90,2.35) | 2.12(200.44) | 1.08(0.91) | 2.11(1.89) |
| venlafaxine | N02CX;N05BX;N06AX | 333 | 2.20(1.97,2.45) | 2.20(215.58) | 1.13(0.97) | 2.19(1.96) |
| clarithromycin | D06AX;J01FA | 285 | 5.32(4.73,5.97) | 5.30(988.09) | 2.40(2.20) | 5.27(4.69) |
| tramadol | N02AX | 267 | 2.56(2.27,2.89) | 2.56(252.43) | 1.35(1.17) | 2.55(2.26) |
| valproic acid | N02CX;N03AG;N05AX | 263 | 2.03(1.80,2.29) | 2.03(136.63) | 1.02(0.83) | 2.02(1.79) |
| furosemide | C03CA | 239 | 3.35(2.95,3.81) | 3.35(391.93) | 1.74(1.54) | 3.34(2.94) |
| fluoxetine | N05BX;N06AB;N07XX | 235 | 2.71(2.38,3.08) | 2.70(250.93) | 1.43(1.23) | 2.69(2.37) |
| mirtazapine | N06AX | 222 | 3.65(3.20,4.16) | 3.64(423.61) | 1.86(1.65) | 3.63(3.18) |
| colchicine | C01EB;L04AX;M04AC | 213 | 20.94(18.28,23.99) | 20.58(3953.93) | 4.36(4.03) | 20.49(17.89) |
| methylprednisolone | D07AA;D07AC;H02AB;S01BA | 205 | 2.72(2.37,3.12) | 2.72(221.82) | 1.44(1.22) | 2.71(2.36) |
| haloperidol | N05AD | 202 | 5.72(4.98,6.57) | 5.69(778.84) | 2.50(2.27) | 5.67(4.94) |
| fluvastatin | C10AA | 191 | 28.71(24.87,33.16) | 28.03(4962.83) | 4.80(4.40) | 27.92(24.18) |
| alprazolam | N05BA | 189 | 2.08(1.80,2.40) | 2.07(104.95) | 1.05(0.83) | 2.07(1.79) |
| azithromycin | D10AF;J01FA;S01AA | 184 | 2.72(2.35,3.15) | 2.72(199.19) | 1.44(1.21) | 2.71(2.35) |
| escitalopram | N05BX;N06AB | 184 | 2.37(2.05,2.73) | 2.36(144.22) | 1.24(1.01) | 2.36(2.04) |
| amiodarone | C01BD | 179 | 2.59(2.23,2.99) | 2.58(172.99) | 1.37(1.14) | 2.58(2.22) |
| lovastatin | C10AA | 174 | 54.45(46.76,63.42) | 51.99(8678.41) | 5.70(5.10) | 51.81(44.49) |
| gemfibrozil | C10AB | 174 | 53.50(45.94,62.31) | 51.12(8527.93) | 5.67(5.08) | 50.94(43.75) |
| paliperidone | N05AX | 173 | 2.28(1.97,2.65) | 2.28(124.30) | 1.19(0.96) | 2.28(1.96) |
| hydroxychloroquine | L04AX;P01BA | 164 | 2.92(2.50,3.40) | 2.91(205.12) | 1.54(1.30) | 2.90(2.49) |
| zolpidem | N05CF | 158 | 2.49(2.13,2.91) | 2.49(140.41) | 1.31(1.07) | 2.48(2.12) |
| diazepam | M03BX;N03AE;N05BA | 141 | 2.78(2.36,3.28) | 2.78(160.32) | 1.47(1.21) | 2.77(2.35) |
| telbivudine | J05AF | 136 | 41.82(35.23,49.64) | 40.36(5210.22) | 5.33(4.72) | 40.25(33.91) |
| terbinafine | D01AE;D01BA;G01AX | 132 | 4.51(3.80,5.36) | 4.50(358.75) | 2.17(1.88) | 4.49(3.78) |
| donepezil | N06DA | 131 | 5.62(4.74,6.68) | 5.60(494.26) | 2.48(2.18) | 5.59(4.71) |
| lorazepam | N03AE;N05BA | 128 | 2.49(2.10,2.97) | 2.49(114.02) | 1.31(1.04) | 2.49(2.09) |
| methadone | N02AC;N07BC | 127 | 4.49(3.77,5.34) | 4.47(341.70) | 2.16(1.86) | 4.46(3.75) |
| linezolid | J01XX | 120 | 3.74(3.12,4.47) | 3.73(239.32) | 1.90(1.60) | 3.72(3.11) |
| diltiazem | C05AE;C08DB | 119 | 3.63(3.03,4.35) | 3.62(225.48) | 1.85(1.56) | 3.62(3.02) |
| abiraterone | L02BX | 118 | 2.04(1.70,2.45) | 2.04(62.52) | 1.03(0.75) | 2.04(1.70) |
| candesartan | C09CA | 115 | 4.39(3.65,5.27) | 4.37(298.64) | 2.13(1.82) | 4.36(3.63) |
| voriconazole | J02AC;S01AX | 114 | 3.56(2.96,4.28) | 3.55(208.57) | 1.83(1.52) | 3.54(2.95) |
| losartan | C09CA | 114 | 2.51(2.09,3.02) | 2.51(103.54) | 1.33(1.04) | 2.51(2.09) |
| fluconazole | D01AC;J02AC;S01AX | 109 | 3.92(3.25,4.73) | 3.91(235.86) | 1.97(1.65) | 3.90(3.23) |
| ticagrelor | B01AC | 109 | 2.39(1.98,2.89) | 2.39(87.90) | 1.25(0.96) | 2.39(1.98) |
| itraconazole | J02AC | 105 | 8.64(7.13,10.47) | 8.58(702.10) | 3.10(2.72) | 8.56(7.06) |
| sevoflurane | N01AB | 103 | 12.52(10.31,15.21) | 12.40(1077.95) | 3.63(3.19) | 12.37(10.19) |
| trabectedin | L01CX | 101 | 28.10(23.06,34.24) | 27.44(2570.21) | 4.78(4.15) | 27.39(22.48) |
| pitavastatin | C10AA | 99 | 21.67(17.76,26.44) | 21.28(1911.26) | 4.41(3.85) | 21.24(17.41) |
| dabrafenib | L01EC | 98 | 2.67(2.19,3.26) | 2.67(102.22) | 1.41(1.10) | 2.67(2.19) |
| hydrochlorothiazide | C03AA | 94 | 3.67(2.99,4.49) | 3.66(181.31) | 1.87(1.53) | 3.65(2.98) |
| diphenhydramine | A04AD;D04A;D04AA;N04AB;N05CM;R06AA | 94 | 2.99(2.44,3.66) | 2.98(123.70) | 1.57(1.25) | 2.98(2.43) |
| allopurinol | M04AA | 91 | 3.17(2.58,3.89) | 3.16(134.18) | 1.66(1.32) | 3.16(2.57) |
| famotidine | A02BA | 89 | 6.51(5.28,8.01) | 6.47(411.54) | 2.69(2.30) | 6.46(5.25) |
| ziprasidone | N05AE | 74 | 2.60(2.07,3.27) | 2.60(72.51) | 1.37(1.01) | 2.59(2.06) |
| oseltamivir | J05AH | 67 | 2.01(1.58,2.56) | 2.01(34.05) | 1.01(0.64) | 2.01(1.58) |
| raltegravir | J05AJ | 64 | 5.41(4.23,6.92) | 5.39(228.89) | 2.43(1.98) | 5.39(4.21) |
| irbesartan | C09CA | 63 | 3.12(2.43,3.99) | 3.11(90.18) | 1.64(1.23) | 3.11(2.43) |
| amfetamine | N06BA | 60 | 4.95(3.84,6.38) | 4.93(187.97) | 2.30(1.84) | 4.93(3.82) |
| piperacillin;tazobactam | J01CR | 59 | 2.49(1.92,3.21) | 2.48(52.20) | 1.31(0.90) | 2.48(1.92) |
| hydrocortisone | A01AC;A07EA;C05AA;D07A;D07AA;D07AB;D07AC;H02AB;S01BA;S02BA | 59 | 2.15(1.66,2.77) | 2.14(35.95) | 1.10(0.70) | 2.14(1.66) |
| erythromycin | D06AX;D10AF;J01FA;S01AA | 58 | 5.28(4.08,6.83) | 5.26(200.00) | 2.39(1.92) | 5.25(4.06) |
| telmisartan | C09CA | 54 | 3.43(2.63,4.48) | 3.42(92.61) | 1.77(1.32) | 3.42(2.62) |
| triheptanoin | A16AX | 52 | 51.15(38.73,67.54) | 48.96(2442.77) | 5.61(4.28) | 48.91(37.04) |
| fenofibric acid | C10AB | 51 | 14.39(10.91,18.96) | 14.22(626.53) | 3.83(3.10) | 14.20(10.77) |
| sirolimus | C01EB;L01EG;L04AH;S01XA | 51 | 2.17(1.65,2.85) | 2.17(32.00) | 1.11(0.68) | 2.16(1.64) |
| memantine | N06DX | 50 | 2.11(1.60,2.79) | 2.11(29.29) | 1.08(0.64) | 2.11(1.60) |
| febuxostat | M04AA | 49 | 5.11(3.86,6.77) | 5.09(161.14) | 2.35(1.82) | 5.09(3.84) |
| verapamil | C08DA;N02CX | 49 | 2.29(1.73,3.03) | 2.29(35.54) | 1.19(0.75) | 2.29(1.73) |
| ranolazine | C01EB | 48 | 2.34(1.76,3.10) | 2.33(36.62) | 1.22(0.77) | 2.33(1.76) |
| hydroxyzine | A04AD;D04A;D04AA;N05BB;R06AE | 47 | 3.94(2.96,5.24) | 3.93(102.56) | 1.97(1.47) | 3.92(2.95) |
| isoniazid | J04AC | 46 | 6.32(4.73,8.45) | 6.29(204.82) | 2.65(2.08) | 6.29(4.71) |
| indapamide | C03BA | 45 | 7.08(5.28,9.49) | 7.04(233.09) | 2.81(2.21) | 7.03(5.24) |
| lopinavir;ritonavir | J05AR | 45 | 2.31(1.73,3.10) | 2.31(33.40) | 1.21(0.74) | 2.31(1.72) |
| entecavir | J05AF | 42 | 4.90(3.62,6.63) | 4.88(129.57) | 2.29(1.72) | 4.88(3.60) |
| meropenem | J01DH | 41 | 3.67(2.70,4.99) | 3.66(79.44) | 1.87(1.34) | 3.66(2.70) |
| ritonavir | J05AE | 41 | 2.17(1.60,2.95) | 2.17(25.81) | 1.12(0.63) | 2.17(1.60) |
| atazanavir | J05AE | 39 | 4.11(3.00,5.63) | 4.10(91.43) | 2.03(1.47) | 4.10(2.99) |
| fluvoxamine | N06AB | 38 | 7.73(5.62,10.63) | 7.68(220.86) | 2.94(2.25) | 7.68(5.58) |
| ketoconazole | D01AC;G01AF;H02CA;J02AB | 38 | 3.58(2.60,4.92) | 3.57(70.41) | 1.84(1.28) | 3.57(2.60) |
| atorvastatin;ezetimibe | C10BA | 36 | 25.95(18.64,36.11) | 25.38(843.35) | 4.66(3.45) | 25.37(18.23) |
| suxamethonium | M03AB | 34 | 17.29(12.32,24.26) | 17.04(513.57) | 4.09(3.05) | 17.03(12.14) |
| rocuronium | M03AC | 34 | 4.62(3.30,6.47) | 4.61(96.04) | 2.20(1.57) | 4.60(3.29) |
| dexmedetomidine | N05CM | 33 | 6.13(4.35,8.63) | 6.10(140.75) | 2.61(1.91) | 6.10(4.33) |
| entacapone | N04BX | 32 | 4.91(3.47,6.94) | 4.89(99.00) | 2.29(1.62) | 4.89(3.45) |
| hydrochlorothiazide;losartan | C09DA | 32 | 3.62(2.56,5.13) | 3.61(60.52) | 1.85(1.24) | 3.61(2.55) |
| encorafenib | L01EC | 32 | 2.83(2.00,4.00) | 2.83(37.75) | 1.50(0.92) | 2.82(2.00) |
| paracetamol;tramadol | N02AJ | 31 | 2.94(2.07,4.18) | 2.93(39.51) | 1.55(0.96) | 2.93(2.06) |
| dolutegravir | J05AJ | 30 | 2.33(1.63,3.33) | 2.33(22.73) | 1.22(0.64) | 2.33(1.63) |
| amphotericin b | A01AB;A07AA;D01AA;G01AA;J02AA;S01AA | 30 | 2.12(1.48,3.03) | 2.11(17.61) | 1.08(0.51) | 2.11(1.48) |
| colchicine;probenecid | M04AC | 29 | 51.93(35.78,75.36) | 49.68(1383.69) | 5.63(3.70) | 49.65(34.21) |
| darunavir | J05AE | 29 | 3.33(2.32,4.80) | 3.33(47.23) | 1.73(1.10) | 3.33(2.31) |
| thiamazole | H03BB | 28 | 5.00(3.45,7.25) | 4.99(89.23) | 2.32(1.59) | 4.98(3.44) |
| galantamine | N06DA | 28 | 3.77(2.60,5.46) | 3.76(56.75) | 1.91(1.24) | 3.76(2.59) |
| cobimetinib | L01EE | 27 | 6.20(4.25,9.05) | 6.17(117.04) | 2.62(1.83) | 6.17(4.23) |
| theophylline | R03DA | 27 | 4.27(2.93,6.24) | 4.26(67.40) | 2.09(1.38) | 4.26(2.92) |
| adefovir | J05AF | 27 | 3.72(2.55,5.43) | 3.72(53.60) | 1.89(1.21) | 3.71(2.55) |
| telithromycin | J01FA | 27 | 2.73(1.87,3.98) | 2.73(29.51) | 1.45(0.81) | 2.72(1.87) |
| chloroquine | L04AX;P01BA | 26 | 10.92(7.42,16.06) | 10.82(231.82) | 3.43(2.43) | 10.82(7.35) |
| glimepiride | A10BB | 25 | 2.74(1.85,4.06) | 2.74(27.59) | 1.45(0.79) | 2.74(1.85) |
| chlorpromazine | A04AD;N05AA | 25 | 10.37(6.99,15.38) | 10.29(209.66) | 3.36(2.35) | 10.28(6.93) |
| baloxavir marboxil | J05AX | 25 | 7.66(5.17,11.36) | 7.62(143.81) | 2.93(2.03) | 7.62(5.14) |
| norepinephrine | C01CA | 25 | 6.15(4.15,9.11) | 6.12(107.12) | 2.61(1.79) | 6.12(4.13) |
| clomipramine | G04BD;G04BE;M09AX;N02BG;N05BX;N06AA | 25 | 5.39(3.64,7.99) | 5.37(88.96) | 2.42(1.63) | 5.37(3.62) |
| cyclobenzaprine | M03BX | 25 | 3.39(2.29,5.03) | 3.39(42.07) | 1.76(1.07) | 3.39(2.29) |
| zonisamide | N03AX | 25 | 2.77(1.87,4.10) | 2.76(28.15) | 1.47(0.80) | 2.76(1.87) |
| chlortalidone | C03BA | 23 | 13.54(8.97,20.43) | 13.39(263.80) | 3.74(2.55) | 13.38(8.87) |
| amlodipine;atorvastatin | C10BX | 23 | 6.54(4.34,9.86) | 6.51(107.33) | 2.70(1.81) | 6.51(4.32) |
| acetazolamide | C03BA;N03AX;R07A;S01EC | 23 | 6.51(4.32,9.81) | 6.48(106.67) | 2.70(1.81) | 6.48(4.30) |
| amantadine | J05AC;N04BB | 23 | 2.55(1.69,3.84) | 2.55(21.59) | 1.35(0.67) | 2.54(1.69) |
| perindopril | C09AA | 23 | 2.25(1.50,3.39) | 2.25(15.96) | 1.17(0.51) | 2.25(1.49) |
| rabeprazole | A02BC | 22 | 2.34(1.54,3.56) | 2.34(16.89) | 1.23(0.54) | 2.34(1.54) |
| alogliptin | A10BH | 21 | 8.88(5.78,13.64) | 8.82(145.61) | 3.14(2.08) | 8.81(5.74) |
| buspirone | N05BE | 20 | 3.69(2.38,5.73) | 3.69(39.16) | 1.88(1.08) | 3.68(2.38) |
| abacavir | J05AF | 20 | 3.21(2.07,4.97) | 3.20(30.28) | 1.68(0.90) | 3.20(2.06) |
| maprotiline | N06AA | 19 | 20.95(13.31,32.99) | 20.59(354.23) | 4.36(2.73) | 20.58(13.07) |
| hydrochlorothiazide;irbesartan | C09DA | 19 | 2.76(1.76,4.34) | 2.76(21.34) | 1.46(0.70) | 2.76(1.76) |
| vecuronium | M03AC | 18 | 15.06(9.46,23.97) | 14.87(232.99) | 3.89(2.44) | 14.86(9.34) |
| doxylamine | N05CM;R06AA | 18 | 3.63(2.29,5.77) | 3.62(34.22) | 1.86(1.01) | 3.62(2.28) |
| carbidopa;entacapone;levodopa | N04BA | 18 | 2.23(1.41,3.55) | 2.23(12.23) | 1.16(0.40) | 2.23(1.40) |
| fluphenazine | N05AB | 17 | 11.54(7.15,18.61) | 11.43(161.88) | 3.51(2.17) | 11.43(7.09) |
| nelarabine | L01BB | 17 | 7.88(4.89,12.70) | 7.83(101.40) | 2.97(1.82) | 7.83(4.86) |
| fosphenytoin | N03AB | 17 | 7.80(4.84,12.58) | 7.76(100.12) | 2.96(1.81) | 7.76(4.81) |
| posaconazole | J02AC | 17 | 2.01(1.25,3.23) | 2.01(8.61) | 1.01(0.25) | 2.01(1.25) |
| isoflurane | N01AB | 16 | 11.78(7.20,19.27) | 11.66(156.09) | 3.54(2.14) | 11.66(7.13) |
| cobicistat;elvitegravir;emtricitabine;tenofovir alafenamide | J05AR | 16 | 3.50(2.14,5.72) | 3.49(28.47) | 1.80(0.91) | 3.49(2.14) |
| phentermine | A08AA | 15 | 2.44(1.47,4.05) | 2.44(12.72) | 1.29(0.44) | 2.44(1.47) |
| triazolam | N05CD | 15 | 3.64(2.19,6.04) | 3.63(28.60) | 1.86(0.92) | 3.63(2.19) |
| dexamfetamine | N06BA | 15 | 2.50(1.51,4.15) | 2.50(13.47) | 1.32(0.47) | 2.50(1.50) |
| norfloxacin | D06AX;G01AA;J01MA;S01AE;S03AA | 14 | 14.75(8.71,25.00) | 14.58(177.14) | 3.87(2.18) | 14.57(8.60) |
| sonidegib | L01XJ | 14 | 6.25(3.69,10.57) | 6.22(61.35) | 2.64(1.46) | 6.22(3.68) |
| benazepril | C09AA | 14 | 3.54(2.09,5.98) | 3.53(25.43) | 1.82(0.85) | 3.53(2.09) |
| colesevelam | C10AC | 14 | 2.00(1.19,3.38) | 2.00(7.02) | 1.00(0.16) | 2.00(1.18) |
| caffeine | C01CX;D11AX;N06BC;R07AB;V04CG | 13 | 9.06(5.25,15.63) | 8.99(92.41) | 3.17(1.74) | 8.99(5.21) |
| famciclovir | J05AB;S01AD | 13 | 6.58(3.81,11.34) | 6.54(61.08) | 2.71(1.45) | 6.54(3.79) |
| imipramine | G04BD;N06AA | 13 | 3.99(2.32,6.88) | 3.98(29.05) | 1.99(0.94) | 3.98(2.31) |
| candesartan;hydrochlorothiazide | C09DA | 13 | 2.37(1.37,4.08) | 2.36(10.24) | 1.24(0.33) | 2.36(1.37) |
| doxepin | D04AX;N05CM;N06AA | 13 | 2.23(1.29,3.84) | 2.23(8.78) | 1.15(0.26) | 2.23(1.29) |
| colchicum autumnale | M04AC;V90 | 12 | 94.19(52.26,169.76) | 86.97(1020.46) | 6.44(2.68) | 86.95(48.24) |
| sodium polystyrene sulfonate | V03AE | 12 | 10.32(5.85,18.22) | 10.24(100.07) | 3.36(1.78) | 10.23(5.80) |
| carbidopa | N04BA | 12 | 2.61(1.48,4.59) | 2.60(11.84) | 1.38(0.41) | 2.60(1.48) |
| benzylpenicillin | D06AX;J01CE;S01AA | 12 | 2.36(1.34,4.16) | 2.36(9.39) | 1.24(0.29) | 2.36(1.34) |
| gadodiamide | V08CA | 12 | 2.22(1.26,3.91) | 2.22(8.03) | 1.15(0.22) | 2.22(1.26) |
| cefditoren | J01DD | 11 | 7.18(3.97,12.99) | 7.14(58.10) | 2.84(1.40) | 7.14(3.94) |
| atracurium | M03AC | 11 | 6.85(3.79,12.39) | 6.82(54.62) | 2.77(1.36) | 6.81(3.77) |
| selumetinib | L01EE | 11 | 5.56(3.08,10.06) | 5.54(40.96) | 2.47(1.17) | 5.54(3.06) |
| atropine | A03BA;C01CX;S01FA;V03AB | 11 | 3.13(1.73,5.65) | 3.12(15.88) | 1.64(0.57) | 3.12(1.73) |
| drotrecogin alfa | B01AD | 11 | 2.59(1.43,4.68) | 2.59(10.73) | 1.37(0.36) | 2.59(1.43) |
| tranexamic acid | A01AD;B02AA;D11AX;R02AX | 11 | 2.32(1.29,4.20) | 2.32(8.27) | 1.21(0.23) | 2.32(1.28) |
| caspofungin | J02AX | 11 | 2.09(1.16,3.78) | 2.09(6.26) | 1.06(0.10) | 2.09(1.16) |
| tigecycline | J01AA | 11 | 2.08(1.15,3.76) | 2.08(6.14) | 1.05(0.10) | 2.08(1.15) |
| peramivir | J05AH | 10 | 22.27(11.91,41.64) | 21.85(199.14) | 4.45(2.04) | 21.85(11.68) |
| cisatracurium | M03AC | 10 | 10.30(5.52,19.19) | 10.21(83.16) | 3.35(1.60) | 10.21(5.48) |
| amoxicillin;clarithromycin;lansoprazole | A02BD | 10 | 10.85(5.82,20.23) | 10.76(88.59) | 3.43(1.64) | 10.76(5.77) |
| repaglinide | A10BX | 10 | 3.46(1.86,6.43) | 3.45(17.42) | 1.79(0.62) | 3.45(1.85) |
| bempedoic acid | C10AX | 10 | 4.96(2.67,9.24) | 4.95(31.51) | 2.31(0.99) | 4.95(2.66) |
| etravirine | J05AG | 10 | 4.54(2.44,8.44) | 4.52(27.45) | 2.18(0.90) | 4.52(2.43) |
| tenofovir alafenamide | J05AF | 10 | 2.52(1.36,4.69) | 2.52(9.16) | 1.33(0.27) | 2.52(1.35) |
| gatifloxacin | J01MA;S01AE;S03AA | 10 | 2.30(1.23,4.27) | 2.29(7.31) | 1.20(0.17) | 2.29(1.23) |
| fosamprenavir | J05AE | 9 | 5.21(2.70,10.02) | 5.19(30.44) | 2.37(0.95) | 5.19(2.69) |
| butalbital;caffeine;paracetamol | N02BE | 9 | 4.70(2.44,9.04) | 4.68(26.06) | 2.23(0.86) | 4.68(2.43) |
| acarbose | A10BF | 9 | 2.52(1.31,4.84) | 2.52(8.22) | 1.33(0.21) | 2.52(1.31) |
| argatroban | B01AE | 9 | 4.53(2.36,8.73) | 4.52(24.68) | 2.18(0.83) | 4.52(2.35) |
| crotalidae polyvalent immune fab | J06AA | 9 | 3.46(1.80,6.66) | 3.45(15.70) | 1.79(0.56) | 3.45(1.79) |
| dactinomycin | L01DA | 9 | 3.42(1.78,6.58) | 3.41(15.37) | 1.77(0.54) | 3.41(1.77) |
| naloxone | A06AH;N07BC;V03AB | 9 | 3.09(1.61,5.95) | 3.08(12.69) | 1.63(0.44) | 3.08(1.60) |
| cerivastatin | C10AA | 8 | 32.77(16.22,66.19) | 31.87(239.36) | 4.99(1.87) | 31.86(15.77) |
| biperiden | N04AA | 8 | 10.70(5.33,21.47) | 10.61(69.68) | 3.41(1.39) | 10.61(5.29) |
| dantrolene | M03CA | 8 | 9.67(4.82,19.41) | 9.60(61.68) | 3.26(1.33) | 9.60(4.79) |
| lovastatin;nicotinic acid | C10BA | 8 | 7.56(3.77,15.15) | 7.51(45.21) | 2.91(1.16) | 7.51(3.75) |
| fosinopril | C09AA | 8 | 6.66(3.32,13.34) | 6.62(38.21) | 2.73(1.06) | 6.62(3.30) |
| metamfetamine | N06BA | 8 | 4.99(2.49,9.99) | 4.97(25.39) | 2.31(0.82) | 4.97(2.48) |
| alogliptin;metformin | A10BD | 8 | 16.32(8.12,32.81) | 16.10(113.42) | 4.01(1.62) | 16.10(8.01) |
| dicycloverine | A03AA | 8 | 4.60(2.30,9.22) | 4.59(22.47) | 2.20(0.75) | 4.59(2.29) |
| nicardipine | C08CA | 8 | 3.27(1.64,6.55) | 3.27(12.59) | 1.71(0.42) | 3.27(1.63) |
| ampicillin;sulbactam | J01CR | 8 | 2.55(1.27,5.09) | 2.54(7.49) | 1.35(0.15) | 2.54(1.27) |
| deucravacitinib | L04AF | 8 | 2.54(1.27,5.09) | 2.54(7.46) | 1.34(0.15) | 2.54(1.27) |
| mefenamic acid | M01AG | 7 | 8.68(4.13,18.26) | 8.62(47.20) | 3.11(1.12) | 8.62(4.10) |
| bempedoic acid;ezetimibe | C10BA | 7 | 4.40(2.09,9.24) | 4.39(18.31) | 2.13(0.60) | 4.38(2.09) |
| maraviroc | J05AX | 7 | 3.54(1.68,7.43) | 3.53(12.71) | 1.82(0.40) | 3.53(1.68) |
| abacavir;lamivudine;zidovudine | J05AR | 7 | 3.13(1.49,6.58) | 3.13(10.13) | 1.64(0.28) | 3.13(1.49) |
| quinine | M09AA;P01BC;R05X | 7 | 2.43(1.16,5.10) | 2.43(5.88) | 1.28(0.02) | 2.43(1.16) |
| danazol | A14AA;G03XA | 6 | 12.42(5.56,27.78) | 12.30(62.33) | 3.62(1.14) | 12.30(5.50) |
| levocarnitine | A16AA;C01EB | 6 | 12.22(5.47,27.32) | 12.10(61.14) | 3.60(1.13) | 12.10(5.41) |
| metformin;pioglitazone | A10BD | 6 | 5.23(2.35,11.67) | 5.21(20.44) | 2.38(0.61) | 5.21(2.34) |
| opicapone | N04BX | 6 | 3.07(1.38,6.85) | 3.07(8.36) | 1.62(0.15) | 3.07(1.38) |
| flucloxacillin | J01CF | 5 | 18.46(7.63,44.67) | 18.18(81.22) | 4.18(1.04) | 18.17(7.51) |
| polymyxin b | A07AA;D06AX;J01XB;S01AA;S02AA;S03AA | 5 | 17.70(7.32,42.82) | 17.44(77.55) | 4.12(1.03) | 17.44(7.21) |
| orphenadrine | M03BC;N04AB | 5 | 15.17(6.27,36.65) | 14.98(65.27) | 3.90(0.98) | 14.97(6.20) |
| perphenazine | N05AB | 5 | 6.16(2.56,14.83) | 6.13(21.49) | 2.62(0.54) | 6.13(2.55) |
| amlodipine;telmisartan | C09DB | 5 | 6.09(2.53,14.66) | 6.06(21.13) | 2.60(0.53) | 6.06(2.52) |
| anidulafungin | J02AX | 5 | 5.35(2.22,12.89) | 5.33(17.62) | 2.41(0.45) | 5.33(2.21) |
| cocaine | A01AD;N01BC;R02AD;S01HA;S02DA | 5 | 4.22(1.75,10.16) | 4.21(12.25) | 2.07(0.27) | 4.21(1.75) |
| lurbinectedin | L01XX | 5 | 4.08(1.70,9.82) | 4.07(11.59) | 2.02(0.25) | 4.07(1.69) |
| dietary supplement | A13A | 5 | 4.49(1.86,10.80) | 4.47(13.50) | 2.16(0.32) | 4.47(1.86) |
| sodium bicarbonate | A01A;A02AH;A16AX;B05BB;B05CB;B05XA;B05Z;D11AX;G02CX;G04BC;G04BX;R01AX;S02DC | 5 | 3.09(1.29,7.44) | 3.09(7.05) | 1.63(0.01) | 3.09(1.28) |
| bromazepam | N05BA | 4 | 11.54(4.31,30.90) | 11.43(38.11) | 3.51(0.59) | 11.43(4.27) |
| esmolol | C07AB | 4 | 7.79(2.91,20.84) | 7.75(23.52) | 2.95(0.42) | 7.75(2.90) |
| trihexyphenidyl | N04AA | 4 | 6.33(2.37,16.92) | 6.30(17.85) | 2.66(0.32) | 6.30(2.36) |
| denileukin diftitox | L01XX | 4 | 5.95(2.23,15.88) | 5.92(16.37) | 2.57(0.28) | 5.92(2.22) |
| safinamide | N04BD | 4 | 5.56(2.08,14.84) | 5.53(14.87) | 2.47(0.24) | 5.53(2.07) |
| tedizolid | J01XX | 4 | 4.33(1.62,11.56) | 4.32(10.20) | 2.11(0.08) | 4.32(1.62) |
| miglitol | A10BF | 4 | 4.67(1.75,12.46) | 4.65(11.47) | 2.22(0.13) | 4.65(1.74) |
| insulin nos | A10A;A10AB;A10AC;A10AD;A10AE;A10AF | 4 | 4.21(1.58,11.23) | 4.19(9.74) | 2.07(0.06) | 4.19(1.57) |
| sodium nitrite | V03AB | 3 | 153.03(45.80,511.29) | 134.78(398.70) | 7.07(0.42) | 134.77(40.34) |
| bezafibrate | C10AB | 3 | 105.21(32.21,343.58) | 96.27(283.09) | 6.59(0.43) | 96.27(29.48) |
| delorazepam | N05BA | 3 | 24.22(7.72,76.02) | 23.73(65.37) | 4.57(0.36) | 23.73(7.56) |
| ritodrine | G02CA | 3 | 16.92(5.41,52.90) | 16.68(44.26) | 4.06(0.30) | 16.68(5.33) |
| ciprofloxacin;fluocinolone acetonide | D07CC;S02CA | 3 | 14.96(4.79,46.74) | 14.78(38.57) | 3.89(0.28) | 14.78(4.73) |
| eravacycline | J01AA | 3 | 14.64(4.69,45.72) | 14.46(37.62) | 3.85(0.27) | 14.46(4.63) |
| amoxicillin;clarithromycin;esomeprazole | A02BD | 3 | 25.90(8.24,81.35) | 25.34(70.18) | 4.66(0.37) | 25.33(8.06) |
| phendimetrazine | A08AA | 3 | 13.47(4.31,42.04) | 13.32(34.21) | 3.74(0.25) | 13.32(4.27) |
| alogliptin;pioglitazone | A10BD | 3 | 6.72(2.16,20.91) | 6.69(14.52) | 2.74(0.02) | 6.69(2.15) |

Notes: MGPS was used only as a supplementary robustness assessment and was not included in the primary signal definition criteria. Drugs associated with multiple ATC codes were assigned to a single first-level ATC category according to their predominant therapeutic indication to avoid duplicate counting. Combination products were analyzed as independent drug entities and classified into one corresponding ATC category.

**Table S7.** Top 20 drugs by reporting frequency of rhabdomyolysis across four therapeutic categories in the FAERS database

| **Drug name** | **Case**  **Reports** | **ROR(95% CI)** | **PRR(χ2)** | **IC(IC025)** | **EBGM**  **(EBGM05)** | **Package insert suggests risk for rhabdomyolysis** |
| --- | --- | --- | --- | --- | --- | --- |
| **Cardiovascular categories** | | | | | | |
| simvastatin | 3731 | 62.67(60.55,64.86) | 60.41(196241) | 5.77(5.70) | 54.45(52.61) | D |
| atorvastatin | 3454 | 23.31(22.51,24.15) | 23.00(65973.5) | 4.39(4.33) | 20.96(20.23) | D |
| rosuvastatin | 2539 | 29.19(28.02,30.40) | 28.68(63234.4) | 4.74(4.67) | 26.79(25.72) | D |
| ezetimibe;simvastatin | 447 | 61.07(55.52,67.16) | 58.73(25075.8) | 5.86(5.55) | 58.03(52.77) | D |
| ezetimibe | 416 | 18.25(16.55,20.11) | 18.04(6624.61) | 4.16(3.96) | 17.85(16.19) | D |
| amlodipine | 308 | 2.47(2.21,2.76) | 2.47(267.10) | 1.30(1.12) | 2.46(2.20) | N |
| fenofibrate | 276 | 24.07(21.36,27.12) | 23.71(5962.38) | 4.56(4.27) | 23.54(20.89) | PM |
| pravastatin | 259 | 20.28(17.93,22.93) | 20.02(4650.61) | 4.31(4.03) | 19.89(17.59) | D |
| furosemide | 196 | 3.65(3.17,4.20) | 3.64(374.18) | 1.86(1.63) | 3.63(3.15) | N |
| gemfibrozil | 149 | 60.41(51.26,71.18) | 58.10(8333.40) | 5.85(5.15) | 57.87(49.11) | DDI |
| lovastatin | 124 | 50.77(42.44,60.74) | 49.14(5832.06) | 5.61(4.88) | 48.98(40.94) | D |
| fluvastatin | 119 | 23.50(19.60,28.17) | 23.15(2515.26) | 4.53(4.02) | 23.08(19.25) | D |
| candesartan | 111 | 5.62(4.66,6.77) | 5.60(418.61) | 2.48(2.15) | 5.59(4.64) | PM |
| amiodarone | 110 | 2.10(1.75,2.54) | 2.10(63.51) | 1.07(0.78) | 2.10(1.74) | PM |
| losartan | 91 | 2.66(2.17,3.27) | 2.66(94.08) | 1.41(1.08) | 2.66(2.16) | PM |
| hydrochlorothiazide | 78 | 4.03(3.23,5.04) | 4.03(177.19) | 2.01(1.63) | 4.02(3.22) | N |
| diltiazem | 77 | 3.11(2.49,3.89) | 3.11(109.90) | 1.63(1.27) | 3.10(2.48) | N |
| pitavastatin | 75 | 21.68(17.26,27.24) | 21.39(1455.49) | 4.42(3.74) | 21.34(16.99) | D |
| irbesartan | 56 | 3.68(2.83,4.78) | 3.67(108.61) | 1.87(1.42) | 3.66(2.82) | N |
| telmisartan | 45 | 3.79(2.83,5.08) | 3.78(92.15) | 1.92(1.41) | 3.78(2.82) | PM |
| **nervous categories** | | | | | | |
| levetiracetam | 998 | 9.84(9.24,10.48) | 9.78(7662.09) | 3.25(3.15) | 9.55(8.96) | N |
| quetiapine | 645 | 3.23(2.99,3.49) | 3.23(974.39) | 1.67(1.55) | 3.19(2.95) | D |
| olanzapine | 582 | 4.58(4.22,4.97) | 4.57(1599.19) | 2.17(2.05) | 4.51(4.16) | PM |
| risperidone | 444 | 2.91(2.65,3.19) | 2.90(547.24) | 1.53(1.38) | 2.88(2.62) | D |
| aripiprazole | 360 | 2.48(2.23,2.75) | 2.47(313.39) | 1.30(1.14) | 2.46(2.22) | D |
| venlafaxine | 310 | 2.72(2.43,3.04) | 2.72(333.51) | 1.43(1.26) | 2.70(2.42) | PM |
| sertraline | 308 | 2.45(2.19,2.74) | 2.45(262.34) | 1.29(1.11) | 2.44(2.18) | PM |
| propofol | 292 | 19.61(17.46,22.02) | 19.37(5049.83) | 4.26(4.01) | 19.22(17.12) | D |
| tramadol | 243 | 3.10(2.73,3.51) | 3.09(342.12) | 1.62(1.42) | 3.08(2.71) | N |
| valproic acid | 219 | 2.24(1.96,2.56) | 2.24(149.97) | 1.16(0.96) | 2.24(1.96) | N |
| fluoxetine | 203 | 3.10(2.70,3.56) | 3.10(287.08) | 1.63(1.41) | 3.09(2.69) | N |
| mirtazapine | 200 | 4.36(3.80,5.02) | 4.35(514.38) | 2.12(1.89) | 4.34(3.77) | PM |
| haloperidol | 196 | 7.37(6.40,8.48) | 7.34(1067.55) | 2.87(2.62) | 7.30(6.34) | PM |
| alprazolam | 178 | 2.60(2.24,3.01) | 2.59(173.76) | 1.37(1.14) | 2.59(2.23) | N |
| paliperidone | 170 | 2.98(2.56,3.47) | 2.98(222.49) | 1.57(1.33) | 2.97(2.55) | D |
| escitalopram | 157 | 2.68(2.29,3.13) | 2.68(164.19) | 1.42(1.17) | 2.67(2.28) | PM |
| zolpidem | 147 | 3.08(2.62,3.62) | 3.07(205.04) | 1.62(1.36) | 3.07(2.61) | N |
| citalopram | 133 | 2.02(1.70,2.40) | 2.02(68.30) | 1.01(0.75) | 2.02(1.70) | PM |
| diazepam | 131 | 3.43(2.89,4.08) | 3.43(224.63) | 1.77(1.50) | 3.42(2.88) | N |
| donepezil | 128 | 7.30(6.13,8.68) | 7.27(689.71) | 2.86(2.53) | 7.24(6.09) | PM |
| **anti-infective categories** | | | | | | |
| daptomycin | 421 | 30.56(27.73,33.67) | 29.97(11662.8) | 4.89(4.65) | 29.64(26.90) | D |
| ciprofloxacin | 271 | 2.23(1.98,2.51) | 2.23(182.41) | 1.15(0.97) | 2.22(1.97) | N |
| clarithromycin | 220 | 5.44(4.76,6.21) | 5.42(789.46) | 2.43(2.21) | 5.40(4.73) | PM |
| azithromycin | 165 | 3.24(2.78,3.78) | 3.24(253.84) | 1.69(1.45) | 3.23(2.77) | N |
| voriconazole | 94 | 3.89(3.18,4.77) | 3.88(200.96) | 1.96(1.62) | 3.88(3.17) | N |
| linezolid | 93 | 3.84(3.13,4.71) | 3.83(194.48) | 1.94(1.60) | 3.83(3.12) | PM |
| fluconazole | 88 | 4.20(3.40,5.18) | 4.19(213.30) | 2.06(1.71) | 4.18(3.39) | DDI |
| Itraconazole | 74 | 8.06(6.41,10.13) | 8.02(454.02) | 3.00(2.54) | 8.00(6.37) | DDI |
| oseltamivir | 63 | 2.51(1.96,3.22) | 2.51(57.15) | 1.33(0.93) | 2.51(1.96) | N |
| telbivudine | 58 | 23.14(17.85,30.00) | 22.80(1208.10) | 4.51(3.68) | 22.77(17.56) | PM |
| piperacillin;tazobactam | 50 | 2.79(2.12,3.69) | 2.79(57.42) | 1.48(1.02) | 2.79(2.11) | D |
| erythromycin | 45 | 5.43(4.05,7.28) | 5.41(161.81) | 2.44(1.88) | 5.41(4.03) | DDI |
| raltegravir | 43 | 4.82(3.57,6.50) | 4.81(129.52) | 2.26(1.71) | 4.80(3.56) | D |
| isoniazid | 41 | 7.48(5.50,10.16) | 7.44(228.57) | 2.89(2.24) | 7.44(5.47) | N |
| rifampicin | 35 | 2.22(1.60,3.10) | 2.22(23.51) | 1.15(0.62) | 2.22(1.59) | N |
| lopinavir;ritonavir | 34 | 2.32(1.65,3.24) | 2.31(25.37) | 1.21(0.67) | 4.24(3.03) | D |
| ketoconazole | 34 | 4.25(3.04,5.95) | 4.24(84.20) | 2.08(1.47) | 2.31(1.65) | N |
| entecavir | 33 | 5.10(3.62,7.18) | 5.09(108.32) | 2.35(1.69) | 5.08(3.61) | N |
| atazanavir | 29 | 4.05(2.81,5.84) | 4.04(66.45) | 2.02(1.35) | 4.04(2.81) | DDI |
| ritonavir | 29 | 2.04(1.41,2.93) | 2.03(15.25) | 1.02(0.45) | 2.03(1.41) | DDI |
| **digestive and metabolic categories** | | | | | | |
| metformin | 397 | 2.52(2.28,2.78) | 2.52(358.94) | 1.32(1.17) | 2.50(2.26) | PM |
| sitagliptin | 117 | 2.18(1.82,2.62) | 2.18(74.83) | 1.12(0.84) | 2.18(1.82) | PM |
| famotidine | 86 | 8.34(6.75,10.32) | 8.30(551.46) | 3.05(2.62) | 8.29(6.70) | D |
| triheptanoin | 50 | 65.16(49.08,86.50) | 62.47(3022.38) | 5.96(4.41) | 62.39(46.99) | N |
| linagliptin | 24 | 2.11(1.42,3.15) | 2.11(14.04) | 1.08(0.44) | 2.11(1.41) | PM |
| glimepiride | 22 | 3.20(2.11,4.86) | 3.20(33.21) | 1.68(0.94) | 3.20(2.10) | N |
| alogliptin | 21 | 11.78(7.67,18.10) | 11.70(205.47) | 3.55(2.36) | 11.69(7.61) | PM |
| rabeprazole | 20 | 2.83(1.82,4.38) | 2.82(23.54) | 1.50(0.75) | 2.82(1.82) | PM |
| phentermine | 15 | 3.24(1.95,5.38) | 3.23(23.15) | 1.69(0.78) | 3.23(1.95) | N |
| amoxicillin;clarithromycin;lansoprazole | 10 | 14.40(7.73,26.85) | 14.27(123.49) | 3.83(1.82) | 14.27(7.65) | DDI |
| atropine | 10 | 3.77(2.03,7.02) | 3.77(20.32) | 1.91(0.72) | 3.77(2.02) | N |
| acarbose | 8 | 2.97(1.48,5.94) | 2.97(10.43) | 1.57(0.32) | 2.97(1.48) | N |
| repaglinide | 8 | 3.67(1.83,7.34) | 3.66(15.49) | 1.87(0.54) | 3.66(1.83) | N |
| alogliptin;metformin | 7 | 18.92(8.98,39.87) | 18.69(117.28) | 4.22(1.51) | 18.69(8.87) | PM |
| levocarnitine | 6 | 16.22(7.25,36.25) | 16.05(84.72) | 4.00(1.25) | 16.05(7.18) | N |
| Dicycloverine | 6 | 4.58(2.05,10.20) | 4.57(16.72) | 2.19(0.50) | 4.57(2.05) | N |
| sodium bicarbonate | 5 | 4.10(1.71,9.87) | 4.09(11.70) | 2.03(0.25) | 4.09(1.70) | N |
| insulin nos | 4 | 5.58(2.09,14.90) | 5.56(14.99) | 2.48(0.25) | 5.56(2.08) | N |
| dietary supplement | 4 | 4.76(1.78,12.71) | 4.75(11.84) | 2.25(0.15) | 4.75(1.78) | N |
| amoxicillin;clarithromycin;esomeprazole | 3 | 34.36(10.94,107.94) | 33.61(94.97) | 5.07(0.41) | 33.61(10.70) | N |

Notes: D, direct warnings or precautions related to rhabdomyolysis or muscle toxicity; PM, postmarketing reports describing rhabdomyolysis or muscle toxicity; DDI, drug-drug interaction warnings associated with increased risk of rhabdomyolysis or muscle toxicity; N, no explicit mention of rhabdomyolysis or muscle toxicity in the official labeling documents. MGPS is merely used as a supplementary method for verifying the robustness of the results, and is not employed to define the primary positive signals (ROR, PRR, and IC).

**Table S8.** Top 50 drugs ranked by reporting frequency of muscle toxicity based on healthcare professional reports in the FAERS database

| **Drug name** | **Case**  **Reports** | **ROR(95% CI)** | **PRR(****χ²)** | **IC(IC025)** |
| --- | --- | --- | --- | --- |
| Atorvastatin* | 4542 | 28.28(27.40,29.18) | 27.24(102330) | 4.61(4.55) |
| Simvastatin* | 3987 | 49.30(47.66,50.98) | 46.15(159337) | 5.38(5.32) |
| Rosuvastatin* | 2682 | 32.19(30.93,33.50) | 30.79(72378.6) | 4.85(4.78) |
| Levetiracetam* | 963 | 6.11(5.73,6.52) | 6.06(3983.22) | 2.57(2.47) |
| Quetiapine* | 621 | 2.91(2.68,3.15) | 2.90(761.35) | 1.52(1.40) |
| Olanzapine* | 531 | 3.33(3.05,3.62) | 3.31(848.22) | 1.72(1.58) |
| Ezetimibe* | 510 | 15.60(14.28,17.04) | 15.26(6719.95) | 3.91(3.75) |
| Risperidone* | 468 | 2.87(2.62,3.14) | 2.86(559.41) | 1.50(1.36) |
| ezetimibe;simvastatin* | 446 | 49.49(44.92,54.52) | 46.05(19473.6) | 5.51(5.23) |
| Daptomycin* | 400 | 15.43(13.97,17.05) | 15.10(5221.70) | 3.90(3.71) |
| Sertraline* | 380 | 2.76(2.49,3.05) | 2.75(420.71) | 1.45(1.30) |
| Propofol* | 317 | 11.27(10.08,12.60) | 11.09(2893.18) | 3.46(3.25) |
| Pravastatin* | 293 | 20.54(18.27,23.08) | 19.93(5239.81) | 4.31(4.05) |
| Ciprofloxacin* | 288 | 2.28(2.03,2.56) | 2.27(204.28) | 1.18(1.00) |
| Venlafaxine* | 286 | 2.10(1.87,2.36) | 2.10(163.35) | 1.06(0.89) |
| Fenofibrate* | 282 | 18.54(16.47,20.88) | 18.06(4519.09) | 4.16(3.91) |
| Tramadol* | 241 | 2.15(1.89,2.44) | 2.14(146.42) | 1.10(0.90) |
| Clarithromycin* | 239 | 3.86(3.40,4.39) | 3.85(501.26) | 1.94(1.73) |
| Furosemide* | 203 | 2.16(1.88,2.48) | 2.16(126.01) | 1.11(0.90) |
| omeprazole | 201 | 2.32(2.02,2.66) | 2.31(148.94) | 1.20(0.99) |
| Fluoxetine* | 194 | 2.37(2.06,2.73) | 2.36(152.10) | 1.24(1.02) |
| Haloperidol* | 188 | 4.29(3.72,4.95) | 4.27(469.03) | 2.09(1.85) |
| levofloxacin | 187 | 2.01(1.74,2.33) | 2.01(94.72) | 1.00(0.79) |
| Colchicine* | 185 | 15.06(13.01,17.43) | 14.74(2362.65) | 3.88(3.56) |
| Mirtazapine* | 181 | 2.96(2.56,3.43) | 2.95(232.89) | 1.56(1.33) |
| Azithromycin* | 174 | 2.15(1.85,2.50) | 2.15(106.68) | 1.10(0.87) |
| Fluvastatin* | 153 | 19.36(16.48,22.75) | 18.83(2576.84) | 4.23(3.84) |
| Zolpidem* | 150 | 2.41(2.05,2.83) | 2.40(122.67) | 1.26(1.01) |
| Lovastatin* | 139 | 51.80(43.57,61.59) | 48.02(6387.61) | 5.58(4.91) |
| Gemfibrozil* | 137 | 46.48(39.07,55.29) | 43.41(5666.92) | 5.44(4.80) |
| esomeprazole | 136 | 2.29(1.93,2.71) | 2.28(98.05) | 1.19(0.93) |
| Telbivudine* | 130 | 28.43(23.85,33.90) | 27.27(3284.71) | 4.76(4.24) |
| Diazepam* | 129 | 2.02(1.70,2.40) | 2.02(66.02) | 1.01(0.74) |
| Donepezil* | 123 | 3.89(3.26,4.65) | 3.88(262.22) | 1.95(1.66) |
| Clonazepam | 121 | 2.15(1.80,2.57) | 2.14(73.84) | 1.10(0.82) |
| Terbinafine* | 120 | 4.02(3.36,4.82) | 4.01(270.27) | 2.00(1.70) |
| Methadone* | 117 | 3.46(2.89,4.15) | 3.45(203.12) | 1.78(1.49) |
| Lorazepam* | 115 | 2.08(1.73,2.50) | 2.08(64.20) | 1.05(0.77) |
| Linezolid* | 111 | 2.38(1.97,2.87) | 2.37(88.15) | 1.24(0.95) |
| Voriconazole | 106 | 2.39(1.97,2.89) | 2.38(84.77) | 1.25(0.95) |
| Diltiazem* | 105 | 2.83(2.34,3.43) | 2.83(123.76) | 1.50(1.19) |
| Itraconazole | 101 | 5.77(4.75,7.03) | 5.73(394.18) | 2.52(2.16) |
| Losartan* | 98 | 2.76(2.27,3.37) | 2.76(109.52) | 1.46(1.14) |
| Trabectedin | 97 | 16.75(13.69,20.49) | 16.35(1396.38) | 4.03(3.52) |
| Ticagrelor | 96 | 2.98(2.44,3.64) | 2.97(125.39) | 1.57(1.25) |
| Candesartan* | 91 | 3.77(3.07,4.64) | 3.76(183.92) | 1.91(1.56) |
| Fluconazole | 89 | 2.67(2.17,3.29) | 2.67(92.74) | 1.41(1.08) |
| Diphenhydramine | 88 | 2.59(2.10,3.19) | 2.58(85.28) | 1.37(1.03) |
| Dabrafenib | 86 | 2.14(1.73,2.64) | 2.13(51.63) | 1.09(0.76) |
| sevoflurane | 85 | 6.88(5.55,8.52) | 6.82(421.77) | 2.77(2.36) |

* indicates that the drug is also ranked among the top 50 by reporting frequency across all reporters in FAERS.

**Table S9.** Top 50 drugs by reporting frequency of muscle toxicity stratified by sex in the FAERS database

| **Female** | | | | | **Male** | | | | | **Not Specified** | | | | |
| --- | --- | --- | --- | --- | --- | --- | --- | --- | --- | --- | --- | --- | --- | --- |
| **Drug name** | **Case reports** | **ROR**  **(95% CI)** | **PRR(χ²)** | **IC(IC025)** | **Drug name** | **Case reports** | **ROR**  **(95% CI)** | **PRR(χ²)** | **IC(IC025)** | **Drug name** | **Case reports** | **ROR**  **(95%CI)** | **PRR(χ²)** | **IC(IC025)** |
| atorvastatin | 1847 | 30.32  (28.89,31.83) | 29.88  (46198.1) | 4.75(4.66) | atorvastatin | 2920 | 22.37  (21.52,23.26) | 21.78  (51632.0) | 4.29(4.22) | atorvastatin | 508 | 33.37  (30.41,36.62) | 32.51  (13918.8) | 4.87(4.66) |
| simvastatin | 1611 | 73.08  (69.37,76.99) | 70.46  (100328) | 6.00(5.87) | simvastatin | 2857 | 46.22  (44.41,48.10) | 43.70  (106600) | 5.29(5.21) | simvastatin | 363 | 70.54  (63.20,78.72) | 66.62  (21743.6) | 5.95(5.56) |
| rosuvastatin | 1232 | 31.41  (29.63,33.30) | 30.92  (33200.8) | 4.85(4.73) | rosuvastatin | 1647 | 22.65  (21.54,23.83) | 22.02  (31047.3) | 4.37(4.28) | rosuvastatin | 330 | 40.16  (35.86,44.99) | 38.87  (11365.2) | 5.18(4.87) |
| levetiracetam | 265 | 6.07  (5.37,6.85) | 6.05  (1101.53) | 2.58(2.37) | levetiracetam | 596 | 7.40  (6.82,8.03) | 7.33  (3190.84) | 2.85(2.71) | levetiracetam | 160 | 9.44  (8.06,11.06) | 9.37  (1158.11) | 3.19(2.88) |
| quetiapine | 248 | 2.56  (2.26,2.90) | 2.56  (231.82) | 1.34(1.15) | quetiapine | 420 | 2.66  (2.41,2.93) | 2.65  (426.20) | 1.39(1.25) | ezetimibe | 95 | 29.86  (24.31,36.69) | 29.11  (2531.20) | 4.84(4.17) |
| sertraline | 247 | 3.97  (3.50,4.50) | 3.96  (539.55) | 1.97(1.77) | olanzapine | 388 | 2.89  (2.61,3.19) | 2.88  (469.75) | 1.51(1.36) | daptomycin | 88 | 22.13  (17.89,27.38) | 21.72  (1709.61) | 4.42(3.81) |
| olanzapine | 234 | 5.07  (4.46,5.77) | 5.06  (752.69) | 2.32(2.11) | ezetimibe | 311 | 16.49  (14.72,18.46) | 16.13  (4369.91) | 4.00(3.76) | amlodipine | 75 | 3.97  (3.16,4.99) | 3.96  (163.39) | 1.97(1.58) |
| ezetimibe;  simvastatin | 214 | 68.03  (59.30,78.05) | 65.57  (13450.3) | 6.02(5.44) | ezetimibe;simvastatin | 238 | 34.79  (30.53,39.65) | 33.21  (7378.91) | 5.04(4.67) | quetiapine | 57 | 3.84  (2.96,4.99) | 3.83  (118.03) | 1.93(1.48) |
| metformin | 195 | 2.91  (2.53,3.35) | 2.91  (241.76) | 1.53(1.31) | pravastatin | 235 | 21.42  (18.80,24.40) | 20.82  (4401.45) | 4.37(4.06) | olanzapine | 56 | 4.46  (3.42,5.80) | 4.44  (147.75) | 2.14(1.67) |
| ezetimibe | 190 | 19.69  (17.05,22.73) | 19.48  (3297.45) | 4.27(3.93) | propofol | 225 | 16.74  (14.66,19.12) | 16.38  (3226.68) | 4.02(3.73) | aripiprazole | 54 | 2.20  (1.68,2.88) | 2.20  (35.03) | 1.13(0.71) |
| daptomycin | 177 | 51.16  (44.03,59.45) | 49.76  (8377.16) | 5.62(5.06) | daptomycin | 211 | 14.79  (12.89,16.95) | 14.50  (2635.50) | 3.85(3.56) | ezetimibe;  simvastatin | 50 | 107.14  (80.03,143.42) | 97.76  (4743.97) | 6.60(4.65) |
| ciprofloxacin | 169 | 2.93  (2.51,3.40) | 2.92  (211.82) | 1.54(1.30) | ciclosporin | 209 | 2.07  (1.80,2.37) | 2.07  (114.07) | 1.04(0.83) | propranolol | 49 | 6.98  (5.27,9.26) | 6.95  (247.12) | 2.78(2.21) |
| venlafaxine | 160 | 2.52  (2.15,2.94) | 2.51  (144.70) | 1.32(1.08) | fenofibrate | 183 | 16.09  (13.89,18.63) | 15.75  (2514.32) | 3.97(3.64) | tramadol | 49 | 3.80  (2.86,5.03) | 3.79  (99.62) | 1.91(1.42) |
| fenofibrate | 143 | 32.62  (27.63,38.51) | 32.05  (4268.62) | 4.99(4.47) | clarithromycin | 171 | 5.83  (5.01,6.78) | 5.79  (673.93) | 2.53(2.26) | venlafaxine | 49 | 4.33  (3.27,5.74) | 4.32  (123.70) | 2.10(1.60) |
| aripiprazole | 135 | 2.25  (1.90,2.66) | 2.25  (92.70) | 1.16(0.90) | omeprazole | 142 | 2.08  (1.77,2.46) | 2.08  (79.51) | 1.05(0.80) | pregabalin | 47 | 2.45  (1.84,3.27) | 2.45  (40.01) | 1.28(0.82) |
| amlodipine | 127 | 2.30  (1.93,2.74) | 2.30  (92.33) | 1.19(0.92) | tramadol | 137 | 2.21  (1.87,2.62) | 2.21  (90.37) | 1.14(0.88) | risperidone | 46 | 2.53  (1.89,3.39) | 2.53  (42.21) | 1.33(0.86) |
| risperidone | 124 | 3.87  (3.24,4.62) | 3.86  (261.33) | 1.94(1.65) | colchicine | 129 | 17.00  (14.27,20.25) | 16.62  (1887.85) | 4.05(3.63) | furosemide | 39 | 2.45  (1.79,3.36) | 2.45  (33.19) | 1.29(0.78) |
| ciclosporin | 117 | 2.10  (1.75,2.51) | 2.09  (66.54) | 1.06(0.78) | haloperidol | 127 | 4.38  (3.68,5.22) | 4.36  (327.95) | 2.12(1.83) | sertraline | 38 | 2.26  (1.64,3.11) | 2.26  (26.42) | 1.17(0.66) |
| furosemide | 109 | 5.56  (4.60,6.71) | 5.54  (403.53) | 2.46(2.13) | telbivudine | 125 | 31.91  (26.66,38.19) | 30.57  (3564.07) | 4.93(4.36) | pravastatin | 35 | 21.62  (15.45,30.24) | 21.22  (670.24) | 4.40(3.27) |
| propofol | 107 | 17.63  (14.57,21.34) | 17.47  (1652.22) | 4.12(3.63) | fluoxetine | 124 | 3.13  (2.62,3.73) | 3.12  (177.74) | 1.64(1.35) | hydroxychloroquine | 31 | 2.39  (1.68,3.41) | 2.39  (24.94) | 1.25(0.68) |
| hydroxychloroquine | 106 | 4.89  (4.04,5.92) | 4.88  (324.98) | 2.28(1.95) | gemfibrozil | 122 | 43.23  (35.99,51.92) | 40.79  (4720.43) | 5.34(4.67) | lovastatin | 31 | 153.80  (105.51,224.19) | 135.07  (4103.20) | 7.07(4.16) |
| valproic acid | 104 | 2.90  (2.39,3.51) | 2.89  (128.06) | 1.53(1.22) | mirtazapine | 119 | 3.44  (2.87,4.12) | 3.43  (204.30) | 1.77(1.48) | methadone | 30 | 4.30  (3.00,6.16) | 4.28  (75.11) | 2.09(1.43) |
| clarithromycin | 93 | 4.71  (3.84,5.78) | 4.70  (269.93) | 2.23(1.87) | methylprednisolone | 110 | 2.35  (1.95,2.84) | 2.35  (84.69) | 1.23(0.93) | mirtazapine | 29 | 6.57  (4.56,9.48) | 6.54  (135.41) | 2.70(1.93) |
| pravastatin | 93 | 17.37  (14.15,21.31) | 17.21  (1413.10) | 4.10(3.57) | azithromycin | 102 | 2.69  (2.21,3.27) | 2.68  (107.30) | 1.42(1.11) | fenofibrate | 28 | 24.00  (16.49,34.93) | 23.51  (600.50) | 4.55(3.18) |
| prednisone | 89 | 2.59  (2.11,3.20) | 2.59  (86.62) | 1.37(1.04) | fluvastatin | 100 | 21.28  (17.44,25.98) | 20.69  (1869.53) | 4.37(3.82) | lidocaine | 24 | 4.55  (3.04,6.80) | 4.53  (65.81) | 2.17(1.40) |
| alprazolam | 88 | 2.60  (2.11,3.21) | 2.60  (86.38) | 1.37(1.04) | diazepam | 95 | 2.72  (2.22,3.33) | 2.71  (102.50) | 1.44(1.12) | buprenorphine | 24 | 2.35  (1.57,3.51) | 2.35  (18.45) | 1.23(0.57) |
| fluoxetine | 88 | 2.76  (2.24,3.40) | 2.76  (98.15) | 1.46(1.12) | furosemide | 91 | 2.29  (1.86,2.81) | 2.28  (65.61) | 1.19(0.87) | propofol | 23 | 12.60  (8.35,19.03) | 12.47  (241.77) | 3.63(2.48) |
| fluvastatin | 85 | 38.77  (31.26,48.09) | 37.96  (3045.95) | 5.24(4.41) | terbinafine | 88 | 4.50  (3.65,5.55) | 4.48  (237.50) | 2.16(1.80) | ciprofloxacin | 22 | 2.75  (1.81,4.19) | 2.75  (24.40) | 1.45(0.75) |
| methylprednisolone | 84 | 3.17  (2.56,3.93) | 3.16  (123.85) | 1.66(1.31) | escitalopram | 87 | 2.19  (1.77,2.70) | 2.18  (55.71) | 1.12(0.80) | clarithromycin | 21 | 4.79  (3.12,7.36) | 4.78  (62.48) | 2.25(1.40) |
| dexamethasone | 81 | 2.64  (2.13,3.29) | 2.64  (82.31) | 1.40(1.05) | methadone | 85 | 4.30  (3.47,5.33) | 4.28  (213.47) | 2.09(1.73) | colchicine | 20 | 19.81  (12.72,30.86) | 19.48  (349.54) | 4.28(2.73) |
| tramadol | 81 | 2.41  (1.93,2.99) | 2.40  (66.19) | 1.26(0.92) | lovastatin | 79 | 39.85  (31.76,50.00) | 37.77  (2823.60) | 5.24(4.36) | thiamazole | 20 | 30.74  (19.69,47.97) | 29.93  (557.43) | 4.90(3.01) |
| escitalopram | 79 | 2.77  (2.22,3.45) | 2.76  (88.56) | 1.46(1.11) | lorazepam | 77 | 2.89  (2.31,3.62) | 2.88  (94.52) | 1.52(1.16) | azithromycin | 19 | 3.03  (1.93,4.75) | 3.02  (25.59) | 1.59(0.80) |
| mirtazapine | 74 | 3.33  (2.65,4.18) | 3.33  (119.89) | 1.73(1.35) | donepezil | 75 | 5.39  (4.30,6.77) | 5.36  (265.72) | 2.42(2.01) | voriconazole | 18 | 4.83  (3.04,7.68) | 4.81  (54.22) | 2.26(1.33) |
| zolpidem | 72 | 3.20  (2.54,4.04) | 3.20  (108.37) | 1.67(1.29) | voriconazole | 70 | 2.61  (2.06,3.30) | 2.60  (68.93) | 1.38(1.00) | sevoflurane | 18 | 7.29  (4.58,11.60) | 7.25  (96.69) | 2.85(1.78) |
| prednisolone | 68 | 2.36  (1.86,3.00) | 2.36  (53.09) | 1.24(0.86) | linezolid | 67 | 2.79  (2.20,3.55) | 2.79  (76.67) | 1.48(1.09) | escitalopram | 18 | 2.19  (1.38,3.48) | 2.19  (11.61) | 1.13(0.38) |
| haloperidol | 67 | 7.40  (5.82,9.41) | 7.37  (367.73) | 2.88(2.40) | fluconazole | 66 | 3.89  (3.05,4.96) | 3.88  (140.65) | 1.95(1.54) | diphenhydramine | 16 | 3.84  (2.35,6.28) | 3.83  (33.37) | 1.93(1.01) |
| citalopram | 66 | 2.06  (1.62,2.63) | 2.06  (35.96) | 1.04(0.66) | allopurinol | 61 | 2.56  (1.99,3.29) | 2.55  (57.50) | 1.35(0.95) | fluconazole | 14 | 5.59  (3.30,9.46) | 5.57  (52.36) | 2.47(1.34) |
| nivolumab | 65 | 2.01  (1.58,2.57) | 2.01  (33.03) | 1.01(0.63) | losartan | 61 | 2.48  (1.93,3.19) | 2.48  (53.60) | 1.31(0.90) | zolpidem | 14 | 2.50  (1.48,4.23) | 2.50  (12.54) | 1.32(0.43) |
| colchicine | 64 | 22.65  (17.70,29.00) | 22.38  (1303.17) | 4.48(3.71) | diltiazem | 60 | 3.50  (2.71,4.51) | 3.48  (106.21) | 1.80(1.37) | famotidine | 14 | 6.85  (4.05,11.60) | 6.81  (69.33) | 2.77(1.54) |
| lovastatin | 64 | 57.18  (44.56,73.38) | 55.42  (3409.80) | 5.79(4.55) | sevoflurane | 54 | 10.94  (8.36,14.32) | 10.79  (479.21) | 3.43(2.80) | sitagliptin | 14 | 3.18  (1.88,5.38) | 3.18  (20.83) | 1.66(0.72) |
| azithromycin | 63 | 2.62  (2.05,3.36) | 2.62  (62.93) | 1.39(0.99) | famotidine | 54 | 7.79  (5.96,10.19) | 7.72  (315.49) | 2.95(2.39) | hydrochlorothiazide | 14 | 8.48  (5.01,14.35) | 8.42  (91.37) | 3.07(1.74) |
| clonazepam | 63 | 2.15  (1.68,2.76) | 2.15  (38.70) | 1.10(0.72) | candesartan | 54 | 3.75  (2.87,4.90) | 3.74  (108.18) | 1.90(1.44) | fenofibric acid | 14 | 25.35  (14.91,43.10) | 24.80  (319.20) | 4.63(2.50) |
| diltiazem | 55 | 4.33  (3.32,5.65) | 4.33  (140.26) | 2.11(1.64) | pitavastatin | 50 | 26.17  (19.73,34.71) | 25.27  (1164.75) | 4.66(3.68) | donepezil | 13 | 4.69  (2.72,8.09) | 4.67  (37.46) | 2.22(1.11) |
| paliperidone | 54 | 3.09  (2.37,4.04) | 3.09  (76.10) | 1.62(1.18) | amfetamine | 48 | 6.56  (4.93,8.71) | 6.51  (223.54) | 2.70(2.13) | abiraterone | 12 | 6.02  (3.41,10.63) | 6.00  (49.87) | 2.58(1.31) |
| amiodarone | 54 | 3.28  (2.51,4.29) | 3.28  (85.31) | 1.71(1.26) | raltegravir | 47 | 4.53  (3.40,6.04) | 4.51  (128.40) | 2.17(1.65) | itraconazole | 12 | 5.62  (3.18,9.91) | 5.59  (45.19) | 2.48(1.24) |
| candesartan | 54 | 5.55  (4.25,7.26) | 5.54  (200.44) | 2.47(1.96) | diphenhydramine | 43 | 2.72  (2.02,3.67) | 2.71  (46.57) | 1.44(0.95) | alprazolam | 12 | 2.10  (1.19,3.71) | 2.10  (6.92) | 1.07(0.15) |
| itraconazole | 53 | 17.05  (13.00,22.35) | 16.89  (790.64) | 4.07(3.31) | trabectedin | 43 | 25.11  (18.52,34.05) | 24.28  (959.71) | 4.60(3.54) | pitavastatin | 12 | 6.53  (3.70,11.53) | 6.50  (55.78) | 2.70(1.39) |
| ticagrelor | 51 | 4.67  (3.55,6.15) | 4.66  (146.20) | 2.22(1.72) | oseltamivir | 41 | 2.12  (1.56,2.88) | 2.11  (24.03) | 1.08(0.59) | methylprednisolone | 11 | 2.45  (1.36,4.44) | 2.45  (9.44) | 1.29(0.29) |
| trabectedin | 49 | 37.11  (27.95,49.26) | 36.36  (1681.42) | 5.18(4.00) | itraconazole | 40 | 4.83  (3.53,6.59) | 4.80  (120.28) | 2.26(1.68) | linezolid | 11 | 2.93  (1.62,5.30) | 2.92  (13.92) | 1.55(0.50) |
| losartan | 47 | 2.87  (2.15,3.82) | 2.86  (56.82) | 1.51(1.04) | ziprasidone | 39 | 2.37  (1.73,3.25) | 2.37  (30.77) | 1.24(0.74) | nicotinic acid | 11 | 7.13  (3.94,12.91) | 7.09  (57.50) | 2.82(1.39) |

**Table S10.** Top 50 drugs by reporting frequency of muscle toxicity stratified by age in the FAERS database

| **＜18** | | **18-44** | | **45-64** | | **≥65** | |
| --- | --- | --- | --- | --- | --- | --- | --- |
| **Drug name** | **Case reports** | **Drug name** | **Case reports** | **Drug name** | **Case reports** | **Drug name** | **Case reports** |
| levetiracetam | 123 | levetiracetam | 505 | atorvastatin | 1531 | atorvastatin | 2596 |
| zoledronic acid | 85 | quetiapine | 297 | simvastatin | 1342 | simvastatin | 2531 |
| propofol | 62 | olanzapine | 251 | rosuvastatin | 816 | rosuvastatin | 1522 |
| sertraline | 55 | atorvastatin | 197 | quetiapine | 231 | ezetimibe | 210 |
| olanzapine | 53 | risperidone | 169 | ezetimibe | 187 | ezetimibe;simvastatin | 190 |
| quetiapine | 37 | propofol | 158 | levetiracetam | 177 | daptomycin | 165 |
| fluoxetine | 37 | rosuvastatin | 157 | olanzapine | 173 | pravastatin | 164 |
| prednisolone | 30 | venlafaxine | 150 | daptomycin | 141 | clarithromycin | 132 |
| sevoflurane | 29 | simvastatin | 141 | fenofibrate | 137 | ciprofloxacin | 128 |
| bupropion | 28 | sertraline | 127 | metformin | 136 | furosemide | 109 |
| triheptanoin | 19 | fluoxetine | 118 | ciclosporin | 134 | fenofibrate | 107 |
| diphenhydramine | 17 | valproic acid | 116 | ciprofloxacin | 127 | olanzapine | 100 |
| voriconazole | 16 | tramadol | 113 | ezetimibe;simvastatin | 124 | fluvastatin | 97 |
| baclofen | 16 | fentanyl | 97 | risperidone | 113 | ciclosporin | 91 |
| isoniazid | 16 | metformin | 90 | aripiprazole | 109 | donepezil | 91 |
| metformin | 16 | mirtazapine | 84 | pravastatin | 109 | risperidone | 82 |
| methylprednisolone | 15 | telbivudine | 83 | paracetamol | 102 | gemfibrozil | 81 |
| rosuvastatin | 14 | alprazolam | 82 | colchicine | 84 | lovastatin | 78 |
| amphotericin b | 13 | escitalopram | 82 | sertraline | 73 | ticagrelor | 74 |
| suxamethonium | 12 | haloperidol | 81 | omeprazole | 71 | sitagliptin | 71 |
| amlodipine | 12 | diazepam | 67 | methylprednisolone | 66 | colchicine | 64 |
| tramadol | 11 | methadone | 63 | clarithromycin | 66 | candesartan | 63 |
| diclofenac | 10 | methylprednisolone | 61 | dexamethasone | 65 | diltiazem | 61 |
| clarithromycin | 10 | paliperidone | 59 | prednisolone | 64 | fluconazole | 60 |
| simvastatin | 10 | clonazepam | 58 | haloperidol | 64 | levetiracetam | 60 |
| nelarabine | 9 | zolpidem | 57 | valproic acid | 62 | mirtazapine | 60 |
| fluvoxamine | 9 | morphine | 55 | propofol | 61 | paroxetine | 60 |
| diltiazem | 9 | dexamethasone | 45 | paliperidone | 59 | azithromycin | 59 |
| benzylpenicillin | 8 | daptomycin | 45 | gemfibrozil | 58 | losartan | 56 |
| selumetinib | 8 | omeprazole | 44 | citalopram | 55 | aripiprazole | 53 |
| terbinafine | 8 | lorazepam | 43 | hydroxychloroquine | 54 | zolpidem | 51 |
| metoclopramide | 8 | diphenhydramine | 40 | fluvastatin | 52 | methylprednisolone | 49 |
| furosemide | 8 | azithromycin | 39 | azithromycin | 49 | itraconazole | 47 |
| lorazepam | 7 | colchicine | 39 | clopidogrel | 47 | pitavastatin | 47 |
| ketorolac | 6 | terbinafine | 38 | amiodarone | 46 | propofol | 46 |
| vecuronium | 6 | linezolid | 37 | trabectedin | 44 | dabrafenib | 43 |
| rocuronium | 6 | furosemide | 33 | diazepam | 42 | terbinafine | 39 |
| chloroquine | 6 | clarithromycin | 32 | lorazepam | 42 | irbesartan | 39 |
| hydroxychloroquine | 6 | ezetimibe;simvastatin | 32 | furosemide | 41 | erythromycin | 36 |
| clindamycin | 6 | ziprasidone | 30 | mirtazapine | 40 | voriconazole | 36 |
| amiodarone | 6 | amfetamine | 29 | hydrochlorothiazide | 40 | haloperidol | 35 |
| theophylline | 5 | ondansetron | 29 | lovastatin | 38 | hydrochlorothiazide | 34 |
| glycopyrronium | 5 | aciclovir | 27 | lansoprazole | 36 | famotidine | 32 |
| atracurium | 5 | hydroxychloroquine | 27 | losartan | 35 | telmisartan | 31 |
| lithium | 5 | sulfamethoxazole;trimethoprim | 27 | linezolid | 33 | hydroxychloroquine | 28 |
| doxepin | 5 | ezetimibe | 27 | allopurinol | 31 | ketoconazole | 26 |
| hydrocodone;paracetamol | 5 | pravastatin | 26 | lamivudine | 29 | febuxostat | 25 |
| maprotiline | 5 | fenofibrate | 25 | candesartan | 29 | trabectedin | 25 |
| norepinephrine | 5 | lidocaine | 24 | hydrocortisone | 28 | paliperidone | 24 |
| atorvastatin | 5 | loperamide | 21 | terbinafine | 28 | galantamine | 22 |

**Table S11.** Top 50 drugs ranked by ROR signal intensity for muscle toxicity in the WHO VigiAccess Database

| **Drug name** | **Case reports** | **ROR(95% CI)** | **PRR(χ²)** | **IC(IC025)** |
| --- | --- | --- | --- | --- |
| dl-methionine;enoxolone;glycine | 4 | 224.34(78.07,644.63) | 193.54(766.67) | 7.60(0.90) |
| pemafibrate | 32 | 223.30(153.76,324.28) | 192.77(6106.80) | 7.59(4.29) |
| cerivastatin | 5027 | 183.12(177.69,188.72) | 163.20(762419) | 7.26(7.17) |
| fenoverine | 41 | 176.42(127.49,244.13) | 156.82(6349.39) | 7.29(4.59) |
| clinofibrate | 3 | 168.25(50.80,557.28) | 150.33(445.32) | 7.23(0.43) |
| glycyrrhiza spp.;paeonia lactiflora | 62 | 138.96(107.04,180.42) | 126.53(7721.27) | 6.98(5.02) |
| clofibrate;inositol nicotinate | 5 | 118.83(47.69,296.07) | 109.62(538.50) | 6.78(1.29) |
| gemfibrozil | 2244 | 113.65(108.81,118.71) | 105.41(226040) | 6.68(6.55) |
| clevudine | 165 | 110.38(94.19,129.35) | 102.41(16548.4) | 6.68(5.76) |
| amlodipine;azilsartan | 17 | 100.17(61.24,163.85) | 93.56(1557.44) | 6.55(3.22) |
| bezafibrate | 406 | 92.16(83.34,101.92) | 86.57(34197.2) | 6.43(6.01) |
| monascus purpureus | 29 | 86.72(59.60,126.20) | 81.73(2313.45) | 6.35(3.93) |
| taltirelin | 3 | 82.48(25.74,264.27) | 77.95(228.05) | 6.28(0.45) |
| telbivudine | 92 | 82.25(66.65,101.50) | 77.75(6967.59) | 6.28(5.10) |
| glycyrrhiza spp. | 3 | 77.90(24.35,249.14) | 73.85(215.73) | 6.21(0.45) |
| dl-methionine;glycine;glycyrrhizic acid | 17 | 75.68(46.45,123.30) | 71.86(1188.43) | 6.17(3.16) |
| atorvastatin;fenofibrate | 4 | 72.84(26.66,199.02) | 69.29(269.40) | 6.11(0.91) |
| cinnamomum cassia;ephedra spp.;glycyrrhiza spp.;paeonia lactiflora;pueraria montana var. lobata;zingiber officinale;ziziphus jujuba | 11 | 72.42(39.50,132.75) | 68.91(736.59) | 6.11(2.52) |
| demethyl podophyllotoxin benzylidene glucoside;podophyllotoxin benzylidene glucopyranoside | 6 | 67.30(29.67,152.69) | 64.27(373.94) | 6.01(1.56) |
| colchicum autumnale | 9 | 66.07(33.86,128.94) | 63.14(550.78) | 5.98(2.19) |
| simvastatin | 7253 | 65.03(63.45,66.64) | 62.42(400783) | 5.84(5.79) |
| ciprofibrate | 84 | 62.34(50.10,77.58) | 59.73(4849.62) | 5.90(4.82) |
| ubenimex | 10 | 59.67(31.69,112.36) | 57.27(553.25) | 5.84(2.34) |
| ezetimibe;simvastatin | 734 | 57.92(53.78,62.38) | 55.68(39099.9) | 5.79(5.58) |
| triheptanoin | 40 | 55.72(40.62,76.43) | 53.63(2066.37) | 5.74(4.09) |
| plitidepsin | 3 | 48.35(15.29,152.84) | 46.77(134.47) | 5.55(0.44) |
| daptomycin | 680 | 46.74(43.29,50.46) | 45.27(29223.5) | 5.49(5.29) |
| acetylsalicylic acid;pravastatin | 5 | 44.94(18.45,109.49) | 43.58(208.13) | 5.45(1.23) |
| trabectedin | 212 | 43.92(38.30,50.36) | 42.62(8599.98) | 5.41(4.95) |
| colchicine;probenecid | 9 | 41.38(21.32,80.29) | 40.22(344.41) | 5.33(2.10) |
| dinitrophenol | 3 | 40.84(12.96,128.72) | 39.71(113.28) | 5.31(0.42) |
| clofibrate | 37 | 39.65(28.59,54.97) | 38.59(1354.98) | 5.27(3.80) |
| fluvastatin | 522 | 38.97(35.72,42.53) | 37.95(18677.9) | 5.24(5.01) |
| angelica acutiloba;atractylodes lancea;bupleurum falcatum;glycyrrhiza spp.;ligusticum officinale;poria cocos;uncaria spp. | 16 | 38.35(23.34,63.03) | 37.36(566.48) | 5.22(2.86) |
| ascorbic acid;caffeine;glucuronamide | 3 | 35.95(11.43,113.09) | 35.08(99.39) | 5.13(0.41) |
| lovastatin | 924 | 35.37(33.12,37.76) | 34.53(29773.5) | 5.09(4.95) |
| chlorpromazine;phenobarbital;promethazine | 12 | 34.84(19.65,61.78) | 34.02(384.79) | 5.09(2.45) |
| atorvastatin | 7517 | 33.67(32.87,34.48) | 32.97(212293) | 4.91(4.87) |
| fenofibrate | 896 | 33.60(31.44,35.92) | 32.85(27390.5) | 5.02(4.87) |
| colchicine;dicycloverine | 6 | 32.99(14.68,74.13) | 32.26(181.84) | 5.01(1.46) |
| pitavastatin | 329 | 31.99(28.67,35.69) | 31.30(9619.98) | 4.96(4.68) |
| glycyrrhiza spp.;ophiopogon japonicus;oryza sativa;panax ginseng;pinellia ternata;ziziphus jujuba | 3 | 30.48(9.71,95.67) | 29.85(83.72) | 4.90(0.40) |
| clenbuterol | 19 | 29.87(18.96,47.06) | 29.27(519.01) | 4.87(2.95) |
| topiroxostat | 5 | 29.33(12.10,71.12) | 28.75(134.03) | 4.85(1.16) |
| caffeine;paracetamol;promethazine;salicylamide | 23 | 29.06(19.23,43.92) | 28.49(610.34) | 4.83(3.14) |
| dihydroergotamine;heparin;lidocaine | 3 | 27.86(8.88,87.35) | 27.33(76.16) | 4.77(0.39) |
| rosuvastatin | 3645 | 27.52(26.62,28.46) | 27.03(87479.7) | 4.70(4.64) |
| tasonermin | 8 | 27.49(13.66,55.35) | 26.98(200.30) | 4.75(1.82) |
| chlorcyclizine | 3 | 27.31(8.71,85.63) | 26.81(74.60) | 4.74(0.39) |
| pravastatin | 943 | 27.09(25.39,28.90) | 26.60(22990.6) | 4.72(4.58) |

**Table S12.** Top 50 drugs ranked by reporting frequency of muscle toxicity in the WHO VigiAccess Database

| **Drug name** | **Case reports** | **ROR(95% CI)** | **PRR(χ²)** | **IC(IC025)** |
| --- | --- | --- | --- | --- |
| Atorvastatin* | 7517 | 33.67(32.87,34.48) | 32.97(212293) | 4.91(4.87) |
| Simvastatin* | 7253 | 65.03(63.45,66.64) | 62.42(400783) | 5.84(5.79) |
| Cerivastatin | 5027 | 183.12(177.69,188.72) | 163.20(762419) | 7.26(7.17) |
| Rosuvastatin* | 3645 | 27.52(26.62,28.46) | 27.03(87479.7) | 4.70(4.64) |
| Gemfibrozil* | 2244 | 113.65(108.81,118.71) | 105.41(226040) | 6.68(6.55) |
| Pravastatin* | 943 | 27.09(25.39,28.90) | 26.60(22990.6) | 4.72(4.58) |
| Lovastatin* | 924 | 35.37(33.12,37.76) | 34.53(29773.5) | 5.09(4.95) |
| Ezetimibe* | 904 | 25.24(23.62,26.96) | 24.81(20453.2) | 4.62(4.48) |
| Fenofibrate* | 896 | 33.60(31.44,35.92) | 32.85(27390.5) | 5.02(4.87) |
| Olanzapine* | 747 | 4.79(4.46,5.15) | 4.78(2213.93) | 2.25(2.13) |
| ezetimibe;simvastatin* | 734 | 57.92(53.78,62.38) | 55.68(39099.9) | 5.79(5.58) |
| Ciclosporin* | 705 | 5.22(4.85,5.62) | 5.20(2376.27) | 2.37(2.25) |
| Propofol* | 696 | 12.86(11.93,13.86) | 12.75(7478.49) | 3.66(3.53) |
| Daptomycin* | 680 | 46.74(43.29,50.46) | 45.27(29223.5) | 5.49(5.29) |
| Risperidone* | 641 | 2.95(2.73,3.18) | 2.94(815.84) | 1.55(1.43) |
| Quetiapine* | 588 | 2.65(2.44,2.87) | 2.65(598.37) | 1.40(1.27) |
| Levetiracetam* | 526 | 5.04(4.62,5.49) | 5.02(1684.73) | 2.32(2.18) |
| Fluvastatin* | 522 | 38.97(35.72,42.53) | 37.95(18677.9) | 5.24(5.01) |
| Prednisone | 520 | 2.22(2.03,2.42) | 2.21(344.21) | 1.14(1.01) |
| Colchicine* | 491 | 20.94(19.15,22.90) | 20.65(9132.77) | 4.36(4.17) |
| Clarithromycin* | 438 | 5.02(4.57,5.51) | 5.00(1397.22) | 2.32(2.17) |
| Haloperidol* | 432 | 6.72(6.11,7.38) | 6.69(2081.19) | 2.74(2.58) |
| prednisolone | 429 | 3.15(2.87,3.47) | 3.15(625.84) | 1.65(1.50) |
| Methylprednisolone* | 416 | 3.42(3.11,3.77) | 3.42(708.03) | 1.77(1.62) |
| Aripiprazole* | 407 | 2.67(2.42,2.95) | 2.67(423.33) | 1.41(1.26) |
| Bezafibrate | 406 | 92.16(83.34,101.92) | 86.57(34197.2) | 6.43(6.01) |
| Amiodarone* | 360 | 4.06(3.66,4.50) | 4.05(823.43) | 2.01(1.85) |
| nivolumab | 340 | 2.15(1.93,2.39) | 2.15(208.63) | 1.10(0.94) |
| pitavastatin | 329 | 31.99(28.67,35.69) | 31.30(9619.98) | 4.96(4.68) |
| Venlafaxine* | 326 | 2.02(1.81,2.25) | 2.02(166.60) | 1.01(0.84) |
| Hydroxychloroquine* | 220 | 2.84(2.49,3.24) | 2.84(261.36) | 1.50(1.30) |
| Trabectedin | 212 | 43.92(38.30,50.36) | 42.62(8599.98) | 5.41(4.95) |
| Ticagrelor | 206 | 4.38(3.82,5.02) | 4.37(534.34) | 2.12(1.90) |
| Mirtazapine* | 205 | 3.23(2.81,3.70) | 3.22(313.30) | 1.68(1.47) |
| fusidic acid | 202 | 25.17(21.90,28.94) | 24.75(4595.77) | 4.63(4.26) |
| Allopurinol | 192 | 2.89(2.51,3.33) | 2.88(235.88) | 1.53(1.30) |
| nicotinic acid | 192 | 2.43(2.11,2.80) | 2.43(161.29) | 1.28(1.06) |
| **Methadone*** | 181 | 2.54(2.19,2.94) | 2.53(167.97) | 1.34(1.11) |
| Trametinib | 179 | 4.06(3.51,4.71) | 4.06(411.43) | 2.02(1.78) |
| Lorazepam* | 175 | 2.21(1.91,2.57) | 2.21(116.18) | 1.14(0.92) |
| Lithium | 174 | 2.57(2.22,2.99) | 2.57(166.73) | 1.36(1.13) |
| suxamethonium | 170 | 14.07(12.09,16.36) | 13.94(2039.01) | 3.80(3.47) |
| Dabrafenib | 168 | 4.18(3.59,4.87) | 4.17(404.65) | 2.06(1.81) |
| **Diazepam*** | 167 | 2.11(1.81,2.46) | 2.11(97.15) | 1.07(0.84) |
| Clevudine | 165 | 110.38(94.19,129.35) | 102.41(16548.4) | 6.68(5.76) |
| Diltiazem* | 164 | 3.37(2.89,3.93) | 3.37(272.47) | 1.75(1.50) |
| ipilimumab | 159 | 2.16(1.85,2.52) | 2.16(98.67) | 1.11(0.87) |
| zidovudine | 142 | 4.48(3.80,5.28) | 4.47(381.52) | 2.16(1.88) |
| abiraterone* | 138 | 2.58(2.18,3.05) | 2.58(133.18) | 1.36(1.10) |
| sitagliptin | 138 | 2.07(1.75,2.44) | 2.06(75.68) | 1.04(0.79) |

* indicates that this drug is listed in the top 50 by reporting frequency reported in the FAERS database; the drugs marked in red indicate that the risks of muscle toxicity are not mentioned in their instructions.


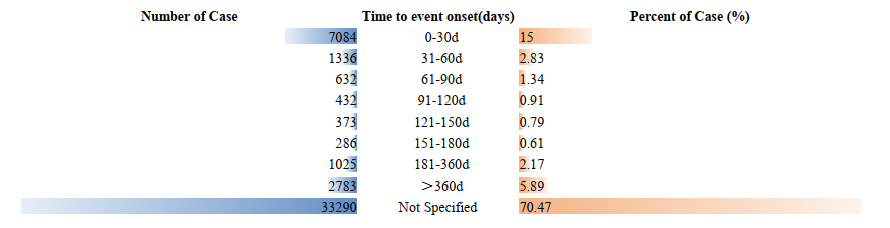


**Figure S1.** Distribution of time to onset (TTO) for muscle toxicity


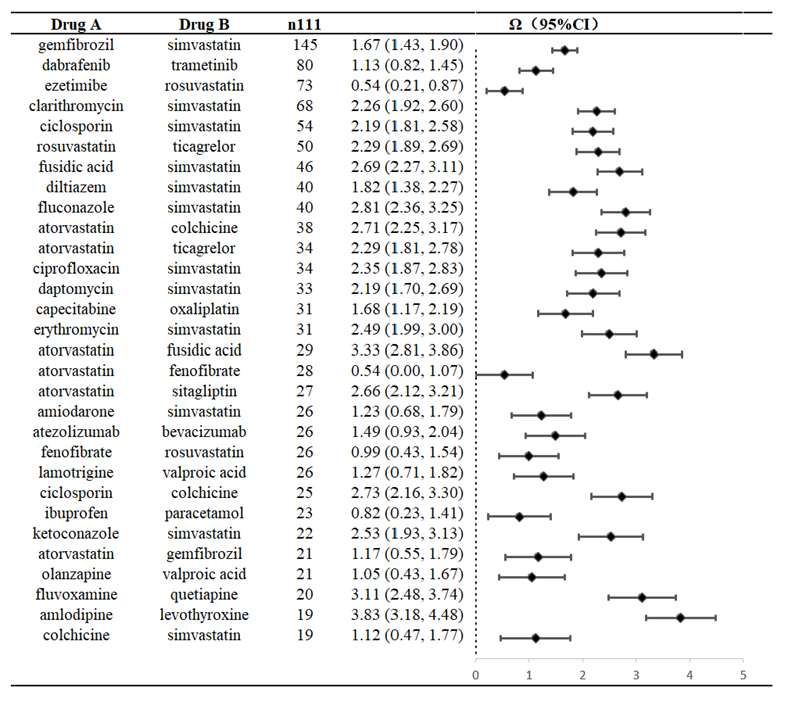


**Figure S2.** Forest plot of Ω values for the top 30 drug pairs with positive signals sorted by the number of reports

**Figure S3.** Distribution of drugs across ATC categories under different minimum report thresholds (n≥10, n≥20, n≥50)

**Figure S4.** Number of drugs with positive signals by therapeutic category in the WHO VigiAccess Database


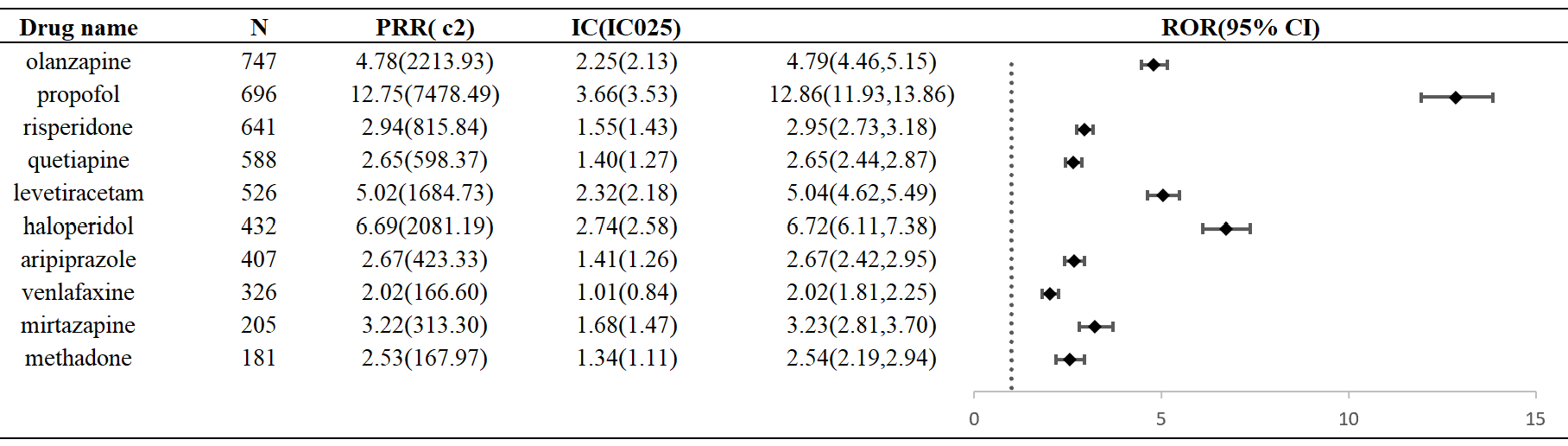


**Figure S5.** Forest plot of ROR signal intensities for the top 10 neurological drugs with signals for muscle toxicity in the WHO VigiAccess Database

Notes: N represents the number of muscular toxicity-related reports for each drug.


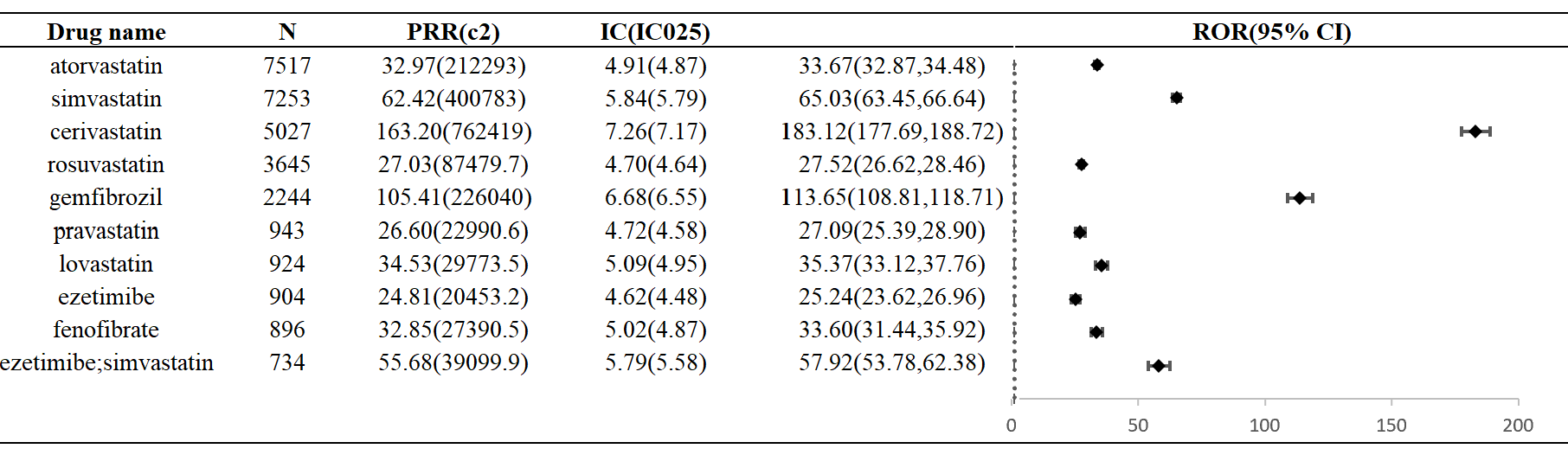


**Figure S6.** Forest plot of ROR signal intensities for the top 10 cardiovascular drugs with signals for muscle toxicity in the WHO VigiAccess Database

Notes: N represents the number of muscular toxicity-related reports for each drug.


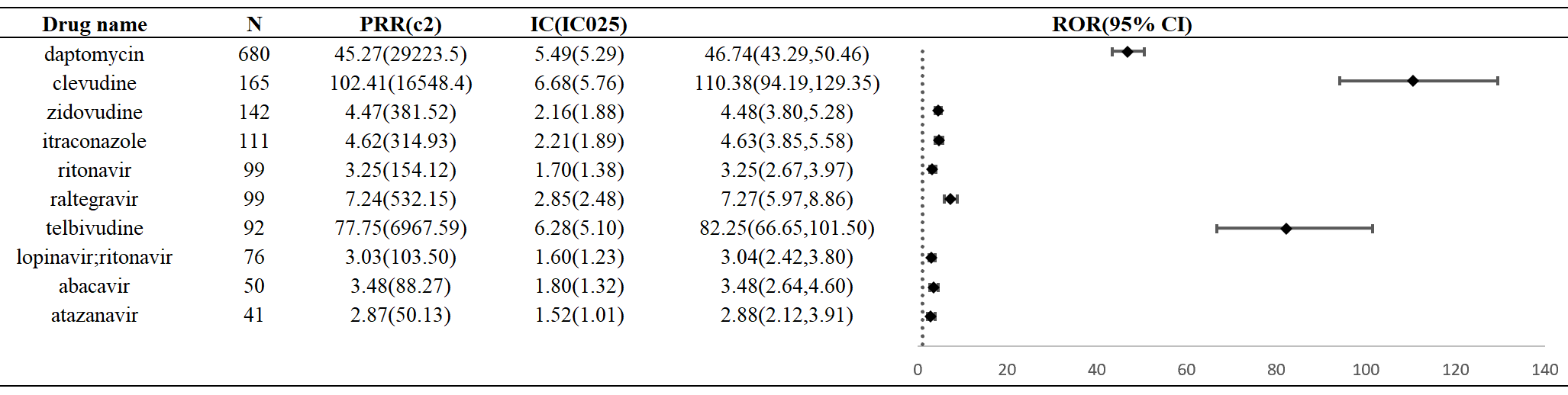


**Figure S7.** Forest plot of ROR signal intensities for the top 10 anti-infective drugs with signals for muscular toxicity in the WHO VigiAccess Database

Notes: N represents the number of muscular toxicity-related reports for each drug.
